# Comparative Genomics of Flowering Time Pathways Using *Brachypodium distachyon* as a Model for the Temperate Grasses

**DOI:** 10.1371/journal.pone.0010065

**Published:** 2010-04-19

**Authors:** Janet A. Higgins, Paul C. Bailey, David A. Laurie

**Affiliations:** 1 Department of Crop Genetics, John Innes Centre, Norwich, United Kingdom; 2 Department of Computational and Systems Biology, John Innes Centre, Norwich, United Kingdom; University of Massachusetts Amherst, United States of America

## Abstract

*Brachypodium distachyon* (Brachypodium) is a model for the temperate grasses which include important cereals such as barley, wheat and oats. Comparison of the Brachypodium genome (accession Bd21) with those of the model dicot *Arabidopsis thaliana* and the tropical cereal rice (*Oryza sativa*) provides an opportunity to compare and contrast genetic pathways controlling important traits. We analysed the homologies of genes controlling the induction of flowering using pathways curated in Arabidopsis Reactome as a starting point. Pathways include those detecting and responding to the environmental cues of day length (photoperiod) and extended periods of low temperature (vernalization). Variation in these responses has been selected during cereal domestication, providing an interesting comparison with the wild genome of Brachypodium. Brachypodium Bd21 has well conserved homologues of circadian clock, photoperiod pathway and autonomous pathway genes defined in Arabidopsis and homologues of vernalization pathway genes defined in cereals with the exception of *VRN2* which was absent. Bd21 also lacked a member of the *CO* family (*CO3*). In both cases flanking genes were conserved showing that these genes are deleted in at least this accession. Segmental duplication explains the presence of two *CO*-like genes in temperate cereals, of which one (*Hd1*) is retained in rice, and explains many differences in gene family structure between grasses and Arabidopsis. The conserved fine structure of duplications shows that they largely evolved to their present structure before the divergence of the rice and Brachypodium. Of four flowering-time genes found in rice but absent in Arabidopsis, two were found in Bd21 (*Id1*, *OsMADS51*) and two were absent (*Ghd7*, *Ehd1*). Overall, results suggest that an ancient core photoperiod pathway promoting flowering via the induction of *FT* has been modified by the recruitment of additional lineage specific pathways that promote or repress *FT* expression.

## Introduction

The switch from vegetative growth (the production of stems and leaves) to reproductive growth (the production of flowers) is an important developmental step in the life cycle of plants. Flowering needs to occur when conditions for pollination and seed development are optimal and consequently most plants restrict flowering to a specific time of year. They commonly achieve this by using reliable environmental cues such as day length (photoperiod) and temperature. In addition, nutrient and water availability and plant size can be important.

The genes and molecular mechanisms controlling flowering have been extensively studied in the model dicot *Arabidopsis thaliana*, subsequently Arabidopsis (reviewed by [1], [2], [3], [4], [5], [6], [7], [8], [9], [10], [11], [12], [13], [14]). As part of this study the Arabidopsis flowering pathways were curated in Arabidopsis Reactome (http://www.arabidopsisreactome.org [15]) to provide an electronic knowledge resource allowing for further developments such as integration with protein-protein interaction datasets, overlaying with microarray data and electronic projection into all newly sequenced plant genomes. Using this we compiled a list of genes and gene families with a known role in flowering time in Arabidopsis.

Flowering time has also been extensively studied in crop species (reviewed by [5], [6], [9], [11], [12], [13], [16], [17], [18], [19]). Flowering time is important for adaptation to specific environments and the world's major crop species provide a particularly interesting opportunity for study because they are grown in areas outside the ecogeographical limits of their wild ancestors. In addition, they are adapted to different farming practices such as fall (autumn) sowing or spring sowing in temperate regions. Adaptation to different environments and practices has been achieved by manipulation of flowering time responses and this makes flowering pathways an excellent system for comparison between and within domestic and wild species.

Comparative studies between Arabidopsis and the tropical cereal rice (*Oryza sativa*) have shown that rice has homologues of many flowering-time genes and that aspects of the photoperiod and autonomous pathway are well conserved (reviewed by [6], [9], [11], [12], [13], [16], [17], [19] Experimental studies have also shown that a gene may retain a role in flowering but with important changes of action. For example the *CONSTANS* (*CO*) gene of Arabidopsis promotes flowering in long days while the equivalent gene in rice (*Hd1* [20]) promotes flowering in short days but represses flowering in long days [9]. In addition, novel flowering-time genes have been found in rice, showing that different plant lineages have evolved new flowering controls. Examples are the rice *Ghd7*, *OsID1*, *Ehd1* and *OsMADS51* genes that are discussed individually in the results section.

Vernalization pathways are significantly different between Arabidopsis and the grasses as the key flowering repressors (*FLC* in Arabidopsis and *VRN2* in cereals) are not conserved (reviewed by [5], [11], [12], [13], [16], [17], [18], [19]). Understanding the control of flowering time in a range of plant species therefore gives us insights into the ancestral control of flowering time and the evolution of alternative mechanisms in different plant lineages.

The wild grass *Brachypodium distachyon* (subsequently Brachypodium) has emerged as an important model for temperate species which include important grain crops such as wheat (*Triticum monococcum*, *T. durum* and *T. aestivum*), barley (*Hordeum vulgare*) and oats (*Avena sativa*) and forage grasses such as *Lolium* and *Festuca* species. The availability of a complete genome sequence for Brachypodium enables the evolutionary relationships of chromosomes to be revealed [21], [22] and is a powerful method for identifying candidates for QTLs identified in individual crop species.

In this paper we used the complete genome sequences of Arabidopsis, rice and Brachypodium to find homologues of genes known to have a role in flowering time in Arabidopsis or other species. For our purposes the genes of interest were those known to affect flowering time measured as leaf number or days to bolting or flowering in Arabidopsis, or as days to panicle emergence, ear emergence or anthesis in cereals. Genes may have been identified from mutation screens or from studies of natural variation. Our aim was to analyse a range of genes involved in known pathways rather than to complete an exhaustive study of all possible flowering-time genes. From this basis phylogenetic analyses of gene families were used to investigate the evolutionary relationships of genes and the impact of segmental duplications on the number of genes in families. Segmental duplications are collinear regions containing paralogous genes that derive from likely whole genome duplication events that occurred in the ancestors of modern species (recently reviewed by [23]). Tandem duplications are instances in which paralogous genes reside side by side along a chromosome and these are likely to result from evolutionarily recent amplification events. The results give us an insight into the evolution of flowering-time genes at the monocot/dicot divide and the relationship between temperate cereals (long-day plants with a vernalization requirement) and tropical cereals (short-day plants with no vernalization requirement).

## Results

**Figure 1:**
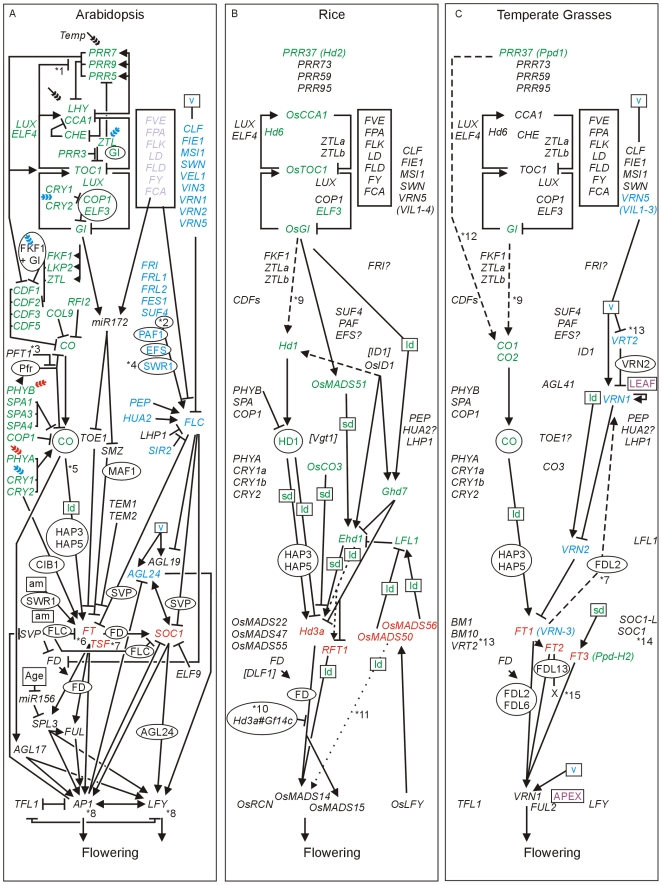
Genetic pathways controlling flowering in Arabidopsis, rice and temperate grasses. Arrows show promoting effects, T-bars show repressing effects. Environmental cues are shown as lower case letters in square boxes; v is extended cold (vernalization); ld is long days; sd is short days; am is ambient (non-vernalizing) temperature. Genes are shown in italics and proteins in non-italics in ovals. Proteins are clearly involved at all stages but we make this distinction only in cases where separate controls are known to exist for transcription and protein function or stability. # indicates inhibition of protein function. The pathways were compiled from the following reviews [1], [2], [3], [4], [5], [6], [7], [8], [9], [11], [12], [13], [14], [16], [17], [18], [19] plus individual articles referenced in the text. Box A. In Arabidopsis genes assigned to pathways are shown in colour (photoperiod pathway in green, vernalization pathway in blue, autonomous pathway in light blue). Flowering pathway integrators are shown in red. Triple headed arrows indicate activation by red or blue light. Box B. Rice genes identified as homologues of those in Arabidopsis are shown in black. Ambiguous homology is indicated with a question mark. Genes with published roles in photoperiod regulated flowering are shown in green and orthologues of pathway integrators in red. Box C. In the temperate grasses box Brachypodium genes identified as homologues of those of Arabidopsis or rice are shown in black. The *VRN2* and *CO3* genes from barley and wheat are also included. Genes with published roles in photoperiod or vernalization regulated flowering in barley or wheat are shown in green or blue, respectively, and orthologues of pathway integrators in red. In B and C likely orthologues of Arabidopsis genes are shown in equivalent positions within the figure (e.g. *Hd1* is the rice orthologue of *CO* and *Hd3a* and *RFT1* are rice orthologues of *FT*). Tables 1 and S1 gives information on gene names and synonyms. Square brackets in box B show three maize genes with known flowering time effects. Notes: *1 TOC1 represses *PRR9*, *PRR7* and *PRR5* [130]. *2 SUF4 interacts with FRI and FRL1. LD blocks SUF4 action to reduce FLC expression [110]. *3 Cerdan and Chory [131] identified *PFT1* and proposed that it promotes flowering by a photoperiod independent pathway activating *FT*. The model here follows Wollenberg et al. [132] who propose an alternative in which *PFT1* acts by repressing Pfr phytochrome signalling. *4 SWR1 is a protein complex including SWC6, ARP6 and PIE1. *5 *CO* expression from phloem-specific promoters complements the *co* mutation, but expression from meristem-specific promoters does not. CO triggers flowering from the phloem by the cell-autonomous activation of *FT* expression [133]. *6 High temperatures (27°C vs 23°C) in short days induce flowering by a mechanism that requires *FT* and gibberellic acid and likely involves *MAF1* (*FLM*). High temperature induction of flowering is independent of the photoperiod pathway but is repressed by FLC and by autonomous pathway mutations that increase FLC levels [122]. Kumar and Wigge [123] showed that ambient temperature responses, including the activation of *FT*, involve the replacement of histone H2A.Z with H2A in nucleosomes. Mutation of ARP6 (part of the SWR1 complex) blocks this process. The late-flowering phenotype of dominant *fwa* mutants is caused by hypomethylation in the *FWA* locus leading to ectopic expression of a homeodomain leucine zipper (HD-ZIP) protein. The FWA protein interacts with FT protein and so ectopic expression of *FWA* delays flowering by impairing FT function [134]. *FWA* is not expressed during the vegetative phase in wild type plants and normally has no role in flowering so homologues are not considered in this paper. *7 *TWIN SISTER OF FT* (*TSF*) is closely related to *FT*, is activated by CO, has a similar role in flowering and is repressed by SVP ([135]; reviewed by [4], [102]). *8 Activation of *AP1* and *LFY* requires two paralogous homeobox genes (*PENNYWISE* (*PNY*; aka *BELLRINGER* and *REPLUMLESS*) and *POUND-FOOLISH* (*PNF*)). Double null mutants express *SOC1* and *FUL* but not *AP1* or *LFY*. The double mutants can receive floral inductive signals but do not properly restructure the shoot apical meristem to make flowers [136], [137]. *9 *GI* promotion of *Hd1*/*CO* is shown directly but could act through the *FKF1*/*CDF* route as in Arabidopsis. *10 Mayfield et al. [80] found that 14-3-3 proteins physically interact with CO in Arabidopsis. *11 It is unclear if there is a direct connection between *OsMADS50* and *OsMADS14*/*15* as well as the connection via *Edh1*. *12 *Ppd-H1* promotion of *CO* is shown directly but could act through the *FKF1*/*CDF* route as in Arabidopsis. *13 Downregulation of *VRT2* by vernalization and a role in repressing *VRN1* is proposed by Kane et al. [107], [108] but is not supported by Trevaskis et al. [109] so *VRT2* is shown in two positions. *14 Shitsukawa et al. [89] found that *WSOC1* was expressed preferentially in leaves. Expression was not affected by photoperiod or vernalization but was upregulated in seedlings by a GA treatment. *15 X indicates an unknown target gene [97]. **Key to figures of phylogenetic trees** A PRANK alignment of protein sequences (shown in the supplementary datasets) was used to construct phylogenetic trees using the neighbor-joining algorithm (subsequently NJ trees) with bootstrap support from 1000 iterations (values of ≥50% shown). Gene identifiers show the species as follows: At for *Arabidopsis thaliana*, Bradi for *Brachypodium distachyon*, Os for *Orzya sativa* (rice), Hv for *Hordeum vulgare* (barley), Ta for *Triticum aestivum* (bread wheat), Tm for *Triticum monococcum* (einkorn wheat), Sb for *Sorghum bicolour* (sorghum) and Zm for *Zea mays* (maize). Barley and wheat proteins are highly homologous and usually only one is shown. Gene names follow the identifiers where relevant to the text and genes with known flowering time roles are shown in colour following Figure 1. In several instances we identified pairs of genes in grasses (paralogues) in segmental duplications (SegDup) which likely result from a grass whole genome duplication that predates the divergence of the rice, maize, sorghum, Brachypodium, barley and wheat lineages. These duplication events are shown as solid circles. Similarly, solid diamonds showduplication events in Arabidopsis; these are independent from the duplication event in the grasses. Boxed T shows tandem or closely arranged paralogues resulting from duplications within individual species. Where the protein belongs to a large family we show small clades containing genes of interest selected from analyses of whole families. The clades were identified either by using an online interrogatory tree of the transcription factor family using known flowering-time genes as query sequences [98] or from the family trees shown in the supplementary material. **Key to figures of segmental duplications** In these figures the size and spacing of genes in the respective genomic sequences is shown to the physical scale indicated in each figure. The components of each half of the duplication are grouped under the bars at the top of the figure. For example, Figure 3 shows part of rice chromosome 3 which is colinear with part of Brachypodium chromosome 1 and is related to part of rice chromosome 7 by segmental duplication. This section of rice 7 is colinear with a separate section of Brachypodium 1. Identifiers and names of flowering-time genes and their homologues are shown, with colours following Figure 1. Gene identifiers are also shown at the top and bottom of each segment to allow orientation to the respective genome browsers. The segmental duplications are recognized by conserved genes (black boxes connected by black lines). Some figures have grey boxes which show genes that are not conserved across the duplication in all species. Within these regions, other genes are conserved between species but not across the duplication (red boxes connected by red lines). These are deletion/insertion events that occurred after the duplication arose but before the divergence of the respective species. Genes in yellow are not conserved across species and represent candidates for deletion/insertion events that occurred after species divergence. Hypothetical genes and sequences annotated as transposons are excluded. In most cases the segmental duplications extend further than shown as we only use those portions that are relevant to the relationships of the flowering-time genes discussed in the text.

The genes and pathways selected are shown in Figure 1 and are listed with names and genomic identifiers in Tables 1 and S1. For clarity, we prefix the gene name with two letters showing the species when more than one species is being discussed: At for *Arabidopsis thaliana*, Bd for *Brachypodium distachyon*, Os for *Orzya sativa* (rice), Hv for *Hordeum vulgare* (barley), Ta for *Triticum aestivum* (bread wheat); Tm for *T. monococcum* (einkorn wheat); Sb for *Sorghum bicolor* (sorghum) and Zm for *Zea mays* (maize). Following Arabidopsis nomenclature, we use italics when referring to genes and non-italic uppercase to refer to proteins in the text.

**Table 1:** Flowering-time genes in Arabidopsis and rice and their Brachypodium homologues.

Gene a	Arabidopsis	Rice	Brachypodium	Cereal	At vs Os b	At vs Bd b	Os vs Bd b	Bd vs cereal^b^	Tree c
*PHYA*	At1g09570	Os03g51030	Bradi1g10520	HvPHYA	63	63	88	88	
*PHYB*	At2g18790	Os03g19590	Bradi1g64360		71	70	93		
*PHYC*	At5g35840	Os03g54084	Bradi1g08400		59	59	88		
*CRY1*	At4g08920	Os02g36380	Bradi3g46590		64	63	85		
*CRY2*	At1g04400	Os02g41550	Bradi3g49200		52	56	80		
*CKA1*	At5g67380	Os03g55389_Hd6	Bradi1g07810		93	80	95		Fig. 2A
*ZTL*	At5g57360	Os06g47890	Bradi1g33610		78	76	92		Fig. 2B
*ELF3*	At2g25930	Os01g38530	Bradi2g14290	TaELF3	25	27	56	78	Fig. 2C
*ELF4*	At2g40080	Os11g40610	Bradi4g13230		33	37	81		Fig. 2D
*GI*	At1g22770	Os01g08700	Bradi2g05230	HvGI	68	66	90	93	
*LHY*	At1g01060	Os08g06110	Bradi3g16515		40	38	73		
*CHE*	At5g08330		Bradi3g60350			50			Fig. S1
*TOC1*	At5g61380	Os02g40510	Bradi3g48880		41	39	79		Fig. 3A
*PRR3*	At5g60100	Os07g49460	Bradi1g16490	HvPpd-H1	36	37	68	71	Fig. 3A
*COP1*	At2g32950	Os02g53140	Bradi3g57670		69	68	88		
*CDF1*	At5g62430	Os03g07360	Bradi1g73710	HvCDF	40	40	75	82	Fig. 4
*CO*	At5g15840	Os06g16370^d^ _Hd1	Bradi1g43670	HvCO1	43	36	70	73	Fig. 5A
*SPA*	At2g46340	Os01g52640	Bradi2g48660		31	41	65		
*TOE1*	At2g28550	Os05g03040	Bradi2g37800	ZmRAP2.7	45	48	69	65	Fig. 6A
*TEM1*	At1g25560	Os01g49830	Bradi2g47220		49	51	73		Fig. 7A
*Ehd1*		Os10g32600							
*ID1*		Os10g28330	Bradi3g26910	ZmID1			57	56	Fig. 7C
*CIB1*	At4g34530								Fig. S4
*FT*	At1g65480	Os06g06320_Hd3a	Bradi1g48830	HvFT1	70	69	86	95	Fig. 8A
*HAP3A*	At2g38880	Os05g38820	Bradi2g22940		73	73	89		Fig. 8B
*HAP5A*	At3g48590	Os03g14669	Bradi1g67980		57	55	81		Fig. 8C
*GF14*υ	At5g16050	Os08g33370	Bradi3g36480		83	82	94		Fig. 9A
*SOC1*	At2g45660	Os03g03070/100^d^	Bradi1g77020	TaSOC1	54	50	61	62	Fig. 9B
*MADS51*		Os01g69850	Bradi2g59190	TaAGL41			66	85	Fig. 9B
*LFL1*		Os01g51610	Bradi2g48060				62		
*LFY*	At5g61850	Os04g51000	Bradi5g20340		51	51	80		
*AP1*	At1g69120	Os03g54160	Bradi1g08340	TaVRN1	52	51	86	86	Fig. 10A
*Ghd7*		Os07g15570							Fig. 11B
*FD*	At4g35900^d^	Os09g36910	Bradi4g36587	ZmDFL1	29	30	48	50	Fig. 12
		Os01g59760	Bradi2g21820	TaFDL2	21	17	62	84	Fig. 12
*SPL*	At2g33810								Fig. S8
*FLC*	At5g10140								Fig.13A
*SVP*	At2g22540	Os03g08754	Bradi1g72150	HvBM1	51	52	72	89	Fig. 13B
*FRI*	At4g00650^d^	Os03g63440	Bradi1g01520		22	19	47		Fig. 13C
*SUF4*	At1g30970	Os09g38790	Bradi4g38000		51	57	76		
*FCA*	At4g16280	Os09g03610	Bradi4g08730	HvFCA	35	35	69	69	
*FY*	At5g13480	Os01g72220	Bradi2g60820		56	57	89		
*FLD*	At3g10390	Os04g47270	Bradi5g18210		69	69	89		
*FVE*	At2g19520	Os01g51300	Bradi2g47940		75	76	90		
*FIE1*	At3g20740	Os08g04270	Bradi3g14520		68	69	87		
*MSI1*	At5g58230	Os03g43890	Bradi1g13930		85	86	94		
*VRN5*	At3g24440	Os12g34850	Bradi4g05950	TmVIL1	40	41	75	82	Fig. 13D
*ARP6*	At3g33520	Os01g16414	Bradi2g10130		64	63	86		
*EMF2*	At5g51230	Os09g13630	Bradi3g03110		50	47	80		Fig. 13E

**a** An expanded list of genes including complete details of gene names, synonyms and accession numbers is provided in Table S1.

**b** ClustalW pairwise alignment scores comparing representative genes for gene families in Arabidopsis (At) rice (Os) Brachypodium (Bd) or a cereal: Barley (Hv), Wheat (Ta) or Maize (Zm).

**c** Please refer to phylogenetic trees in figure listed.

**d** UniProt sequences used for the following loci: Os06g16370 (Q30DN4_ORYSI), Os03g03070/100 (MAD50_ORYSJ), At4g35900 (FD_ARATH), At4g00650 (Q52S96_ARATH).

Brachypodium homologues were identified by BLASTP and TBLASTN searches using Arabidopsis, rice, barley or cereal predicted proteins. The approaches and stringencies are described in full in the materials and methods section. Putative orthologues in Brachypodium were confirmed by reciprocal BLAST searches (Table S1). In cases where flowering-time genes and their close homologues belonged to large gene families, searches were performed using profile Hidden Markov Models (HMMs) [24]. Homologous proteins were aligned using the Probabilistic Alignment Kit (PRANK) [25] which recognises insertions and deletions as individual evolutionary events and takes into account the evolutionary distances between sequences. Phylogenetic trees for each family were estimated from these alignments using distance matrix methods.

It is well known that the Arabidopsis, rice and Brachypodium genomes contain segmental duplications as a result of whole genome duplication events in relatively recent ancestors [26]. The genes residing in these regions may exhibit some functional redundancy which is important to recognise in the present study. We used information on flowering-time genes from the Plant Genome Duplication Database (http://chibba.agtec.uga.edu/duplication) and the segmental duplications function of Rice annotation 6.1 (http://rice.plantbiology.msu.edu/). We also carried out additional TBLASTN searches to compare genomic sequences. Paralogues arising from segmental duplications are shown in the phylogenetic tree diagrams. Paralogues that are tandem duplications on individual chromosomes are shown separately on the phylogenetic trees because they are a result of more recent and localised duplication events and the genes are more likely to be functionally redundant.

As an additional check against rice or, where available, other cereals, flanking genes around the gene of interest were also identified and compared. This provided a genomic context that helped support the orthology of individual genes. Where genes were not found in Brachypodium, the presence of conserved flanking genes and intervening continuous sequence provided evidence that the gene was deleted.

### 1. Light signalling pathways

#### 1.1 Photoreceptors

Light perception by the plant is primarily by the PHOTOTROPIN (PHOT) and CRYPTOCHROME (CRY) blue light receptors and the PHYTOCHROME (PHY) red/far red light receptors. *PHOT* genes do not have a known flowering role and were not analysed. Brachypodium *CRY* genes were similar to genes previously described in rice, with two *CRY1* like genes and one *CRY2* like gene (Table 1). The ZEITLUPE (ZTL) protein is also a blue light receptor (see section 1.2). Three phytochrome genes (*PHYA*, *PHYB* and *PHYC*) were found in Brachypodium, as in rice. Three additional *PhyA* loci are known in barley from RFLP mapping but these may be pseudogenes [27]. This is supported by the absence of the additional copies in Brachypodium. Comparison of flanking genes showed the *PhyA* gene in Brachypodium to be colinear to the rice *PhyA* gene and the likely functional copy on the barley and wheat group 4 chromosomes.

#### 1.2 Circadian clock components

Light and temperature inputs entrain the circadian clock, allowing plants to coordinate metabolic pathways that need to be attuned to the day. The circadian clock also provides the time piece against which day length is measured, allowing flowering to be induced by specific inductive day length conditions (reviewed by [3], [4], [7], [14]).

In Arabidopsis, two genes encoding related myb transcription factors (*CIRCADIAN CLOCK ASSOCIATED 1* (*CCA1*) and *LATE ELONGATED HYPOCOTYL* (*LHY*)) are expressed in the morning and *PSEUDO-RESPONSE REGULATOR 1* (*PRR1*, also called *TOC1*) is expressed in the evening, forming a feedback loop. These three genes form the central oscillator of the circadian clock (Figure 1). The Arabidopsis *CCA1* and *LHY* genes have a single counterpart in rice (*OsCCA1* [28]) and Brachypodium (called *BdCCA1* here). A single homologue of *PRR1*(*TOC1*) is present in Brachypodium, rice and barley. The *PRR1*-like genes could clearly be recognized as an outgroup of the *PRR* gene family. All *PRR1* homologues had a conserved 6 exon structure and were the only *PRR* genes lacking an intron in the CCT domain.

**Figure 2:**
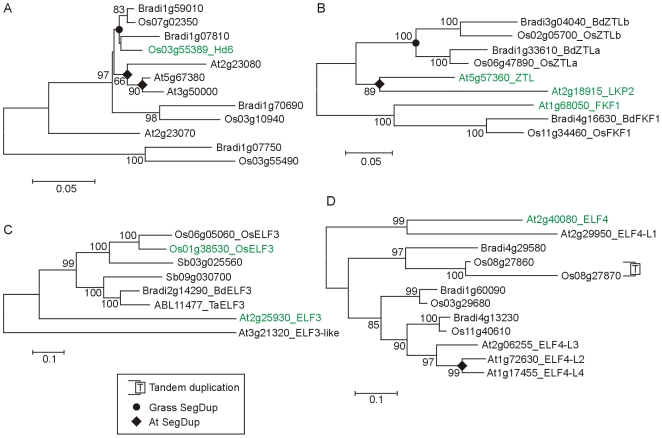
Phylogenetic relationships within gene families containing circadian clock associated proteins. A) NJ tree of Heading date6 (Hd6) and related CASEIN KINASE IIα proteins (whole protein alignment; Dataset S2). B) NJ tree of ZEITLUPE and related proteins (whole protein alignment; Dataset S3). C) NJ tree of EARLY FLOWERING 3 and related proteins (whole protein alignment; Dataset S4). D) NJ tree of EARLY FLOWERING 4 and related proteins (whole protein alignment; Dataset S5).

In rice, the *Heading date6* (*Hd6*) gene was identified as the α subunit of *CASEIN KINASE 2* (*CK2*) which is thought to have a role in CCA1 protein regulation by phosphorylation [29]. The Brachypodium orthologue of *Hd6* was identified as Bradi1g07810. Paralogous genes exist in rice and Brachypodium as a result of segmental duplication (Figure 2A).

CCA1 HIKING EXPEDITION (CHE) interacts with TOC1 to repress *CCA1* [30]. CHE is a TCP family protein. There are 11 high homology matches in Brachypodium to this protein but the amino acid sequence outside the TCP domain was conserved sufficiently between CHE and the Bradi3g60350 protein to suggest that the latter protein was a Brachypodium orthologue (Figure S1).

In Arabidopsis, the circadian clock has additional feedback loops. A morning loop contains members of the *PRR* family and an evening loop contains *EARLY FLOWERING 3* (*ELF3*), *EARLY FLOWERING 4* (*ELF4*), *LUX ARRHYTHMO* (*LUX*) and *GIGANTEA* (*GI*) (Figure 1 [3], [7], [14], [31], [32]). CYCLING DOF FACTOR (CDF), CONSTITUTIVE PHOTOMORPHOGENIC 1 (COP1), ZTL and LOV KELCH PROTEIN 2 (LKP2) proteins all interact with these feedback loops. *ZTL*, the highly homologous *LKP2* and the closely related *FLAVIN-BINDING*, *KELCH REPEAT*, *F BOX 1* (*FKF1*) gene encode ubiquitin-ligase proteins. The COP1 and ELF3 proteins interact and regulate GI by promoting the degradation of GI protein in the dark [33]. Interactions between GI and other proteins allow different functions to be performed. In the circadian clock, the *ZTL* gene is constitutively expressed but blue light enhanced interaction between clock controlled GI and ZTL is required to stabilize the latter. This provides circadian oscillation of ZTL that directs the degradation of TOC1 [34]. In the photoperiod pathway, blue light enhanced interaction of GI and FKF1 are involved in the induction of *CONSTANS* (*CO*) expression (see section 1.3). Arabidopsis *ZTL*, *LKP2* and *FKF1* also act to regulate CDF transcription factors (section 1.3; Figure 1 [35]).

*GI* is a highly conserved single copy gene in Arabidopsis, rice and Brachypodium (where it has a proven roles in flowering time [36], [37] and in barley (Table 1). Rice and Brachypodium have two paralogous *ZTL*-like genes with similar homologies to *AtZTL* and the closely related *AtLKP2*. This suggests independent gene duplication in the monocot and dicot lineages after divergence. One additional gene in rice and Brachypodium was more similar to *AtFKF1* (Figure 2B). Arabidopsis *COP1* had a single orthologue in rice and Brachypodium (Table 1). Arabidopsis *ELF3* and *SIMILAR TO ELF3* (At3g21320) identified two rice genes both of which were most closely related to *AtELF3* (Figure 2C). At least one rice gene has a role in flowering as an Os01g38530 loss of function mutation causes late flowering in short or long days [38]. Two genes were also found in sorghum, one of which was in the same sub-group as the two rice genes. One Brachypodium homologue was found, but flanking genes surrounding the *OsELF3*-like genes did not identify the same Brachypodium location. This suggests that two genes were originally present in grasses, one of which was lost from the temperate grasses. The other gene was lost from rice and the remaining rice gene was subsequently duplicated.

Overall, the results suggest that *GI* and its various interactors are well conserved but that independent duplications affect the *ZTL*/*LKP2* homologues giving the same numbers of genes in Arabidopsis and grasses by different routes.

Two other Arabidopsis genes (*ELF4* and *LUX*) that are involved in the regulation of *CCA1* and *LHY* were also investigated. A single orthologue of the Arabidopsis *LUX* gene was identified in rice and Brachypodium (Table 1). Four rice and three Brachypodium genes were found with homology to Arabidopsis *ELF4* gene, but homology was closest to related members of the Arabidopsis gene family and no convincing orthologue of *ELF4* was identified (Figure 2D).

**Figure 3:**
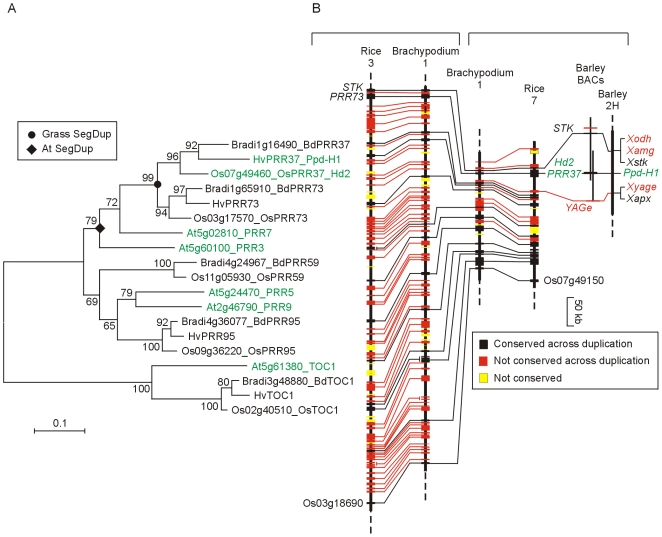
Phylogenetic relationship of PRR proteins. A) NJ tree of PSEUDO-RESPONSE REGULATOR proteins based on alignment of the PSEUDO-RECEIVER (PF00072) and CCT (PF06203) domains; Dataset S6; Full length Barley sequences; Dataset S1). B) Structure of a segmental duplication originally defined in rice using RFLP markers [138] that contains the *PRR37* (*Ppd-H1*, *Hd2*) and *PRR73* genes.

The *Pseudo-Response Regulator* (*PRR*) family consists of five genes in Arabidopsis (*PRR1* (*TOC1*), *PRR3*, *PRR5*, *PRR7* and *PRR9*) and five *PRR* genes in rice (*OsPRR1*, *OsPRR37*, *OsPRR73*, *OsPRR59*, *OsPRR95*) [28]. Five genes were found in Brachypodium and these were closely related to the respective rice genes (Figure 3A). The *PRR1* (*TOC1*) genes were clearly recognizable across all species but the relationships of the remaining grass and Arabidopsis genes was less clear, as reflected in the rice nomenclature.

*PRR37* and *PRR73* are paralogues arising from a segmental duplication (Figure 3B). The gene content of the two segments is consistent with a model in which duplication occurred and experienced a period of gene loss so that the majority of the genes remaining were present in one copy. The derived chromosomes were largely stable and retained a well conserved gene order during the divergence of the major grass lineages. All the duplicated regions we examined were consistent with this model, but the example in Figure 3B was unusual in that the rice 7 region and its Brachypodium equivalent contained many fewer genes, suggesting preferential gene loss from one of the duplicated segments.

*PRR37* and *PRR73* are most similar to *AtPRR7*. *PRR37* is also known as *PHOTOPERIOD 1* (*Ppd1*) as mutations in this gene provide important flowering time variation in barley (*Ppd-H1* [39]) and wheat (*Ppd-A1*, *Ppd-B1*, *Ppd-D1* [40], [41]). *PRR37* in rice has been identified as the *Heading date 2* (*Hd2*) locus [42]. The paralogue (*PRR73*) is not known to provide flowering time variation in any of these species. This may reflect subfunctionalization, and the genes are known to have different expression profiles in rice [43]. *AtPRR7* is thought to have roles in clock function and photoperiod response (Figure 1A), but the cereals genes may have partly separated these functions, allowing photoperiod response to be manipulated by mutation of the *PRR37* gene without compromising clock function.

The two other *PRR* genes in grasses are more similar to *AtPRR5* and *AtPRR9* (Figure 3A). The relationship between these genes is not clear and a further ambiguity is that *AtPRR5* has six exons and *AtPRR9* has seven while the cereal *PRR59* and *PRR95* genes have the same eight exon structure as *OsPRR37*, *OsPRR73* and *AtPRR7*. Therefore, while the *PRR* family has the same number of genes in Arabidopsis and grasses, they may have achieved this number by different routes and the genes in grasses do not relate to the Arabidopsis genes in a simple one to one manner.

#### 1.3 Photoperiod pathway genes

In Arabidopsis the *CONSTANS* (*CO*) gene plays a key role in photoperiod response (reviewed by [9]), but other members of the family are also likely to be involved in flowering (e.g. *COL5* [44]). *CO* is characterized by two B-boxes (a class of zinc-finger domain) near the amino-terminus and a CCT domain at the COOH-terminus. Its transcription is regulated by outputs from the circadian clock and peaks late in the day. *CO* transcription at the start of the day is directly repressed by binding of the Dof transcription factor CDF1 [45]. *CDF1* transcription is repressed by *PRR* genes [46] and the CDF1 protein is removed from the *CO* gene by a blue light activated complex of GI and FKF1 proteins [47]. In this way *GI* acts as a positive regulator of *CO* expression. *CO* expression is also repressed by *CDF2*, *CDF3* and *CDF5* [35]. *CDF2* is known to be regulated by *FKF1* and the related *ZTL* and *LKP2* genes [35]. The activity of the CO protein is also regulated. In short days the expression peak occurs in the dark where CO protein is rapidly degraded. PHYB and SUPPRESSOR OF PHYA-105 (SPA) proteins remove CO protein at the start of the day. If the expression peak coincides with light, which occurs in long days, the CO protein is stabilized by a mechanism involving PHYA and CRY proteins and can then activate *FLOWERING LOCUS T* (*FT*) (section 2.1 [48], [49]).

**Figure 4:**
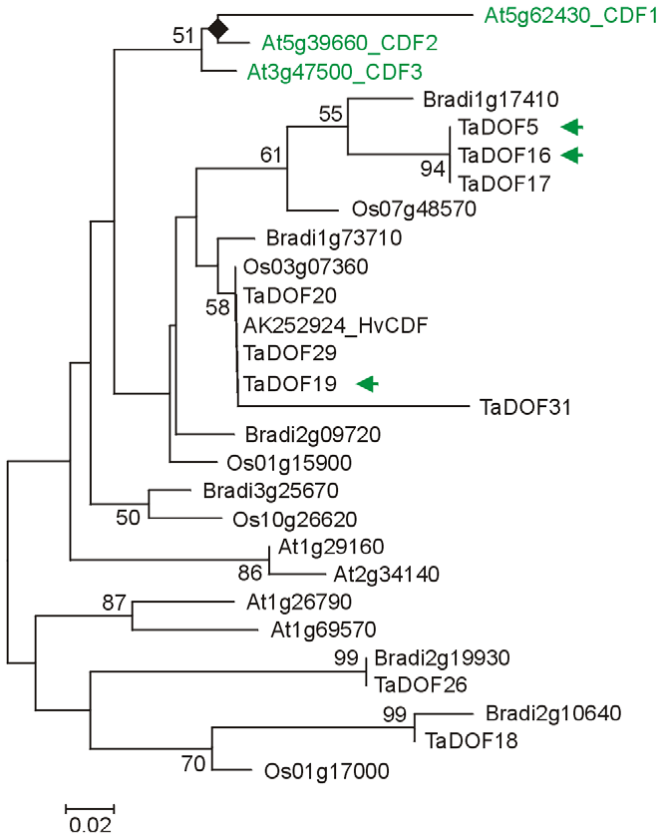
Phylogenetic relationship of CDF proteins. NJ tree of CYCLING DOF FACTOR proteins (whole protein alignment; Dataset S7). The CDF subgroup was identified with an online interrogatory tree using known Arabidopsis flowering-time genes as query sequences [93]. Wheat DOF proteins were taken from Shaw et al. [139] and include three (green arrows) whose expression patterns resembled that of Arabidopsis CDFs involved in flowering.

Four Brachypodium and four rice genes were most closely related to *AtCDF1*, *2* and *3* but the grass and Arabidopsis genes formed separate subgroups (Figure 4) suggesting that the *CDF* family has increased in size after monocot/dicot divergence. Conserved domains were highly conserved between all these proteins while other regions were diverged. Therefore, the relationships between them were ambiguous and a role for individual *CDF* genes in the photoperiod pathway in grasses would need to be established by experimental approaches.

**Figure 5:**
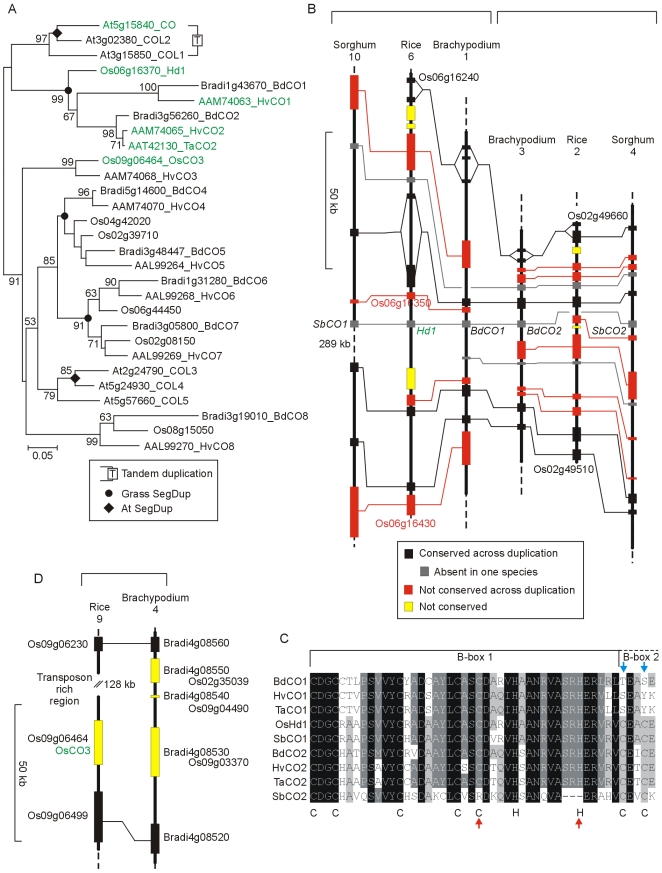
Phylogenetic relationship of CO proteins and physical maps of the *CO1* and *CO2* gene regions. A) NJ tree of CONSTANS and related proteins (whole protein alignment; Dataset S8). This diagram does not contain the diverged group IV CO-like proteins defined by Griffiths et al. [51] as these are described in Figure 11. B) Structure of the segmental duplication containing the *CO1* and *CO2* genes of the grasses. C) Amino-acid sequences of B-box1 and the start of B-box2 from rice, barley, Brachypodium and sorghum proteins. Blue arrows – conserved C residues not present in B-box 2 of the temperate species. Red arrows – conserved C and H residues missing from B-box 1 of the sorghum CO2 protein. Conserved amino acids defining B-boxes are shown below. D) Colinear segments of rice chromosome 9 and Brachypodium chromosome 4 showing the absence of the *CO3* gene from Brachypodium.

*CONSTANS* (*CO*) is a member of a gene family [50] which can be divided into four sub-groups based on conserved domains [51]. The gene family has the same overall structure in Brachypodium as previously shown in rice and barley (Figure 5A [51]). However, barley was previously shown to differ from rice in having two highly *CO*-like genes (*HvCO1* on chromosome 7H and *HvCO2* on chromosome 6H). Barley *HvCO1* is colinear with the rice *CO* homologue *Heading date 1* (*Hd1*) which is an important source of flowering time variation in rice [20]. The wheat *TaCO2* gene (*TaHd1*) is implicated in flowering control as it can complement the rice *hd1* mutation [52].

Brachypodium has two highly *CO*-like genes and comparison with rice shows that these are paralogues arising from segmental duplication. Barley and Brachypodium have retained both copies of the gene while the *HvCO2*/*BdCO2* equivalent has been lost from rice (Figure 5B). The presence of the *HvCO2*/*BdCO2* gene is not specific to temperate grasses because an orthologous gene is present in sorghum (*SbCO2*). However, *SbCO2* is unusual in grasses in lacking highly conserved C and H residues from B-box 1 (red arrows, Figure 5C) suggesting it may be non functional. Griffiths et al. [51] showed that HvCO1 lacks conserved C residues at the start of B-box 2 and this is conserved in Brachypodium (blue arrows, Figure 5C) suggesting that temperate grasses have a CO1 protein with a modified or non-functional second B-box.

The barley *HvCO3* gene and its rice counterpart (*OsCO3*; Os09g06464, a known flowering-time gene [53]) are unusual in having a single B-box that is likely to have arisen from a two B-box ancestor by internal deletion [51]. No homologue of *HvCO3* was found in Brachypodium. Genes flanking *OsCO3* had clear orthologues on Brachypodium chromosome 4 which were separated by three additional genes with different rice locations, suggesting *BdCO3* may have been lost by a complex rearrangement (Figure 5D). Other *CO* family genes were well conserved between Brachypodium, barley and rice. The *CO4* and *CO5* genes are paralogues arising from segmental duplication as are the *CO6* and *CO7* genes (Figure 5A).

In Arabidopsis, SUPPRESSOR OF PHYA-105 (SPA) proteins interact with the CO protein to reduce its stability, inhibiting flowering in short days (Figure 1). Mutations in *SPA1* cause early flowering under short-day (SD) but not long-day (LD) conditions and this is independent of *PHYA*. Mutations of *SPA1*, *SPA3* and *SPA4* in combination have an enhanced phenotype and flower at the same time in LD and SD [48]. The *spa1* mutation is also reported to have an effect on the circadian clock, slightly shortening the circadian period of *CCA1*, *TOC1* and *SPA1* transcript accumulation under constant light conditions [54].

Rice and Brachypodium each had three genes with homology to *AtSPA*. One was the *COP1* orthologue which has a similar WD repeat domain to SPA in the C-terminal region of the protein and which also has a role in degrading CO protein [55]. The related Os05g49590 and Bradi2g15900 genes lacked a protein kinase domain found in AtSPA1,3 and 4 so that only one rice and one Brachypodium gene (Os01g52640 and Bradi2g48660; Table 1) were identified as likely orthologues of AtSPA.

**Figure 6:**
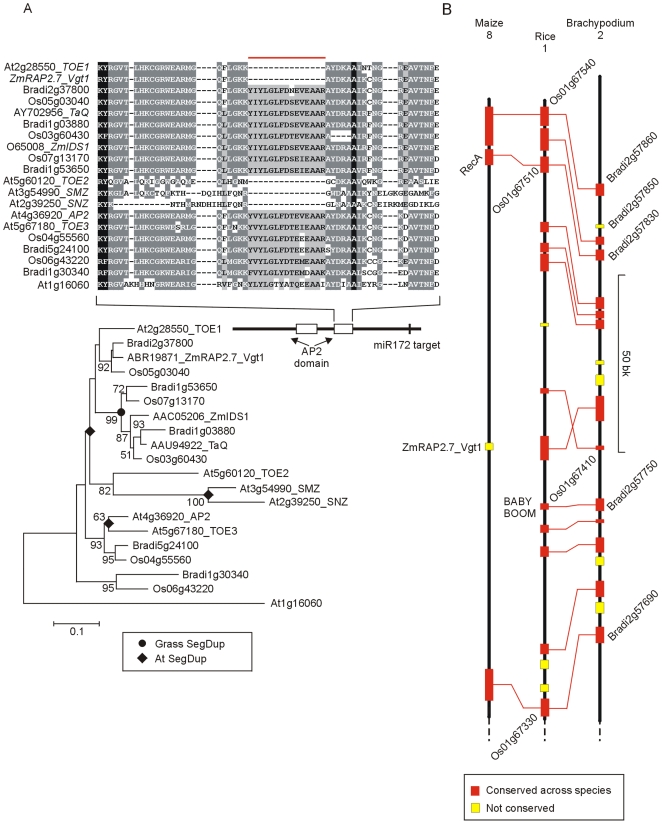
Phylogenetic relationship of TOE1 and related AP2 family genes a physical maps of the maize *Vgt1* region. A) NJ tree of TARGET OF EAT1 and related proteins (whole protein alignment; dataset S9; clade selected from Figure S2 using outgroup root At1g16060). Sequence alignment shows a 15 amino acid deletion in TOE1 and Vgt1 (red bar) that was not found in any Brachypodium or rice gene. The position of the conserved miRNA172 target site is also shown. B) Position of *Vgt1* on maize chromosome 8 and colinear regions of rice chromosome 1 and Brachypodium chromosome 2 showing the absence of *Vgt1* homologues from rice and Brachypodium.

In addition to activation by *CO*, *FT* is regulated by *GI* through an independent pathway in Arabidopsis where GI upregulates microRNA172 which acts as a repressor of the *APETALA 2* (*AP2*) domain gene *TARGET OF EAT1* (*TOE1*) which in turn encodes a repressor of *FT* [56]. *TOE1* is a member of a gene family characterized by two AP2 domains. Other members include *APETALA 2* itself, involved in inflorescence development. The TOE1 subfamily members have a smaller first AP2 domain due to an internal deletion of 10 amino acids and all contain a miR172 target site. Genes of this subfamily are present in grasses (Figure 6A) and include the *Q* gene of wheat and the *INDETERMINATE SPIKELET 1* (*IDS1*) gene of maize, both affecting inflorescence structure [57]. However, TOE1 is unusual in having a deletion of 15 amino acids in the second AP2 domain. No gene with this deletion was found in Brachypodium or rice but the maize *VEGETATIVE 1* (*RAP2.7_Vgt1*) gene, which affects flowering time [58], has the 15 amino-acid deletion (Figure 6A). It is unlikely that identical deletions would occur independently in Arabidopsis and maize so it is possible that this type of gene has been lost from the rice/Brachypodium lineage. Genes flanking *Vgt1* in maize were found on rice chromosome 1 and Brachypodium chromosome 2 (Figure 6B). These regions contained a distantly related AP2 domain gene related to Arabidopsis *BABYBOOM* that is too dissimilar to *Vgt1* to appear in the clade of AP2 domain proteins shown in Figure 6A.

TOE2 and SCHLAFMUTZE (SMZ) also have diverged second AP2 domains. SMZ is an additional factor repressing *FT*, and this repression requires the MADS-box transcription factor *MADS AFFECTING FLOWERING 1* (*MAF1*) gene [59]. No homologue of *SMZ* was identified in rice or Brachypodium (Figure 6A) nor was a homologue of *MAF1* found (see section 5).

**Figure 7:**
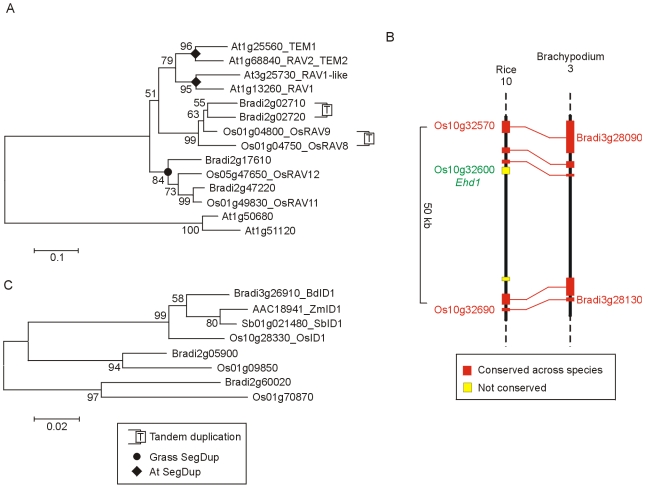
Phylogenetic relationships of TEM, ID1 and Ehd1 proteins. A) NJ tree of TEMPRANILLO and related proteins (whole protein alignment; Dataset S10). The TEM subgroup was identified with an online interrogatory tree using known Arabidopsis flowering-time genes as query sequences [98]. B) Colinear segments of rice chromosome 10 and Brachypodium chromosome 3 showing the absence of *Ehd1* from Brachypodium. C) NJ tree of INDETERMINATE1 proteins (using an alignment of the ID domain; Dataset S11). Two related IDD proteins are included to show that ID proteins are a distinct subgroup of the C2H2 zinc-finger transcription factor family (Figure S3).

In Arabidopsis, *TEMPRANILLO 1* and *2* (*TEM1 and TEM2*) have been identified as additional repressors of *FT* [60]. TEM proteins have one AP2 domain and a B3 domain. *TEM*-like genes were found in rice and Brachypodium but no clear orthologues of *TEM1* or *TEM2* were found (Figure 7A). This suggests that *TEM1* and *TEM2*, with two *RAV* genes, may have originated by gene duplication after the monocot/dicot divergence.

*Early heading date* (*Ehd1*) is a B-type response regulator identified in rice which has no counterpart in Arabidopsis [61]. Its role is to upregulate *Hd3a* (*FT*) in inductive conditions (short days) and it acts independently of *Hd1*(*CO*) (Figure 1B). No orthologue was identified in Brachypodium, although flanking genes were well conserved (Figure 7B), or in temperate grass ESTs suggesting that this is likely to be an additional flowering component that evolved within the rice lineage or was deleted from the temperate grasses.

The rice MADS-box transcription factor *OsMADS51* is regulated by OsGI and upregulates *Ehd1* in short days, providing an alternative route for the upregulation of *Hd3a* (*FT*) in inductive conditions (Figure 1B [62]). *OsMADS51* has no clear Arabidopsis orthologue but in contrast to *Ehd1* there were Brachypodium and wheat (*AGL41*) orthologues. The relationship of the OsMADS51 group to other MADS-box proteins is shown in Figures S7 and 9B. The lack of an *Ehd1* gene in temperate grasses suggests either that the *OsMADS51* orthologue has a different function or that there are additional targets linking it to flowering control.

*INDETERMINATE 1* (*ID1*) was first identified in maize (*Zea mays*) where loss of function mutation causes extremely late flowering [63]. *ID1* encodes a C2H2 zinc-finger transcription factor which has no clear Arabidopsis orthologue. In rice, loss of function mutations of the *ID1* orthologue (*RID1*; Os10g28330, also called *Early heading date2* (*Ehd2*)) show extremely late flowering under short- or long-day conditions [64], [65], [66]. The expression of *ID1* and *RID1* is independent of photoperiod and is necessary for the expression of genes such as *Ehd1* and *Ghd7* involved in regulating the switch from vegetative to floral development (Figure 1B). A phylogenetic tree was constructed for ID1-like proteins in rice and Brachypodium which form a distinct grass specific clade of the C2H2 proteins (Figure 7C). In Brachypodium Bradi3g26910 was identified as the closest equivalent by sequence and the presence of flanking genes. The *BdID1* genomic sequence had a 1 bp insertion giving a frame shift mutation. This could be a feature of the Bd21 accession or a sequencing error.

In Arabidopsis, *FT* expression is also promoted independently of CO by CRYPTOCHROME-INTERACTING BASIC-HELIX-LOOP-HELIX 1 (CIB1) in a blue light dependent interaction with CRY2 [67]. Weak homologies to CIB1 were found in rice and Brachypodium in the bHLH domain but no convincing orthologue could be identified (Figure S4)

### 2. Flowering pathway integrators

#### 2.1 The FLOWERING LOCUS T (FT) family

The induction of *FT* expression is a key step in flowering. Long-day and short-day plants differ in how *FT* expression is regulated but in both cases flowering is associated with increased *FT* expression, suggesting that *FT* is a conserved activator of flowering (reviewed by [9]). In Arabidopsis, FT protein is a mobile signal that moves from leaf to apex where it interacts with the FD protein to activate the *APETALA 1* (*AP1*) gene and initiate the transition to reproductive growth [9], [68], [69]. *FT* and its close homologue *TWIN SISTER OF FT* (*TSF*) are members of a phosphatidylethanolamine binding domain (PEBP) gene family that is more extensive in grasses [70], [71].

**Figure 8:**
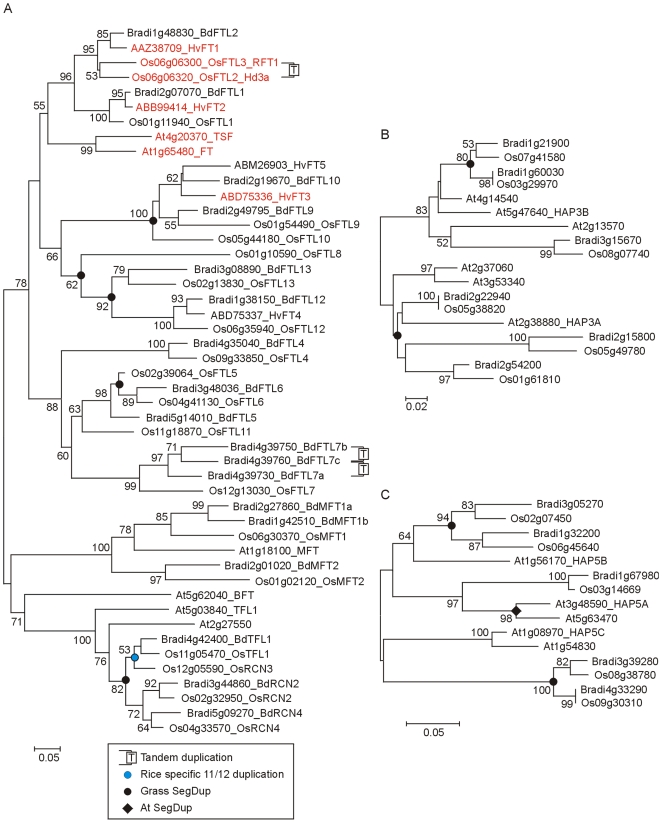
Phylogenetic relationship of FT and HAP family proteins. A) NJ tree of FLOWERING LOCUS T and related proteins (PEBP (PF01161) domain alignment; Dataset S12). B) NJ tree of HAP3 and related proteins (whole protein alignment; Dataset S13). C) NJ tree of HAP5 and related proteins (whole protein alignment; Dataset S14). In B and C only the HAP3 and HAP5 clades selected from Figure S5 are shown.

The clade of PEBP proteins most similar to FT and TSF is encoded by two sub groups of genes in the grasses (Figure 8A). The first comprised *OsFTL1* and its orthologues *BdFTL1* and *HvFT2*. The second comprised two rice genes (*OsFTL2* and *OsFTL3*) that are arranged in tandem and are likely to be a recent duplication so that both can be regarded as orthologues of *HvFT1* and *BdFTL2*. The rice numbering follows Chardon and Damerval [70] and we have also used this for orthologous Brachypodium genes. *OsFTL2* (*Hd3a*) and *OsFTL3* (*RFT1*) promote flowering [72], [73], [74]. *HvFT1* and *HvFT2* are also likely to be flowering promoters because they can act as such in rice [75] but *HvFT1* was thought to be the most likely orthologue of *FT* because it is upregulated at the floral transition [71].

Generally, other rice and Brachypodium genes grouped as recognizable orthologues. However, rice *OsFTL8* and *11* had no counterpart in Brachypodium. *HvFT5* had no detectable expression in barley and may be a pseudogene [71]. It had no counterpart in Brachypodium. The *HvFT3* gene, which is a candidate for the *Ppd-H2* flowering-time locus [71], [75] was present in Brachypodium. The *OsFTL7* orthologue in Brachypodium was present as a tandem triplication (Figure 8A).

In Arabidopsis the *TERMINAL FLOWER 1* (*TFL1*) gene is important in regulating meristem determinacy. Three *TFL1*-like genes were found in Brachypodium. Two had rice orthologues while the third was orthologous to two rice genes known to be part of a recent segmental duplication involving rice chromosomes 11 and 12 [76]. The rice *MOTHER OF FT 1* (*OsMFT1*) gene has two counterparts in Brachypodium (Figure 8A). The Brachypodium *FT*/*TFL* family was therefore similar to barley and rice with differences attributable to the fate of genes in segmental duplications and to different tandem duplications.

#### 2.2 The HEME ACTIVATOR PROTEIN (YEAST) HOMOLOG (HAP) family

The CO protein is thought to act as a transcription factor but has no direct DNA binding activity. Recently it has been shown to form a complex with proteins similar to yeast HAP proteins and it is this complex that activates *FT* expression in Arabidopsis [77]. HAP proteins are present as large families in cereals (Figure S5 [18]) and there were several possible counterparts for the HAP3 and HAP5 proteins involved in CO interaction in Arabidopsis (Figures 8B and 8C, respectively). This suggests diversification of the HAP family following monocot/dicot divergence and further clarification of the relationships will require experimental approaches. The HAP family is of interest in grasses because key photoperiod (Hd1, Ppd-H1) and vernalization (VRN2) proteins belong to the CCT family (sections 1.3 and 3.2) and are likely to act in complexes with HAP proteins. Variation in HAP proteins or complexes could therefore provide functional variation.

**Figure 9:**
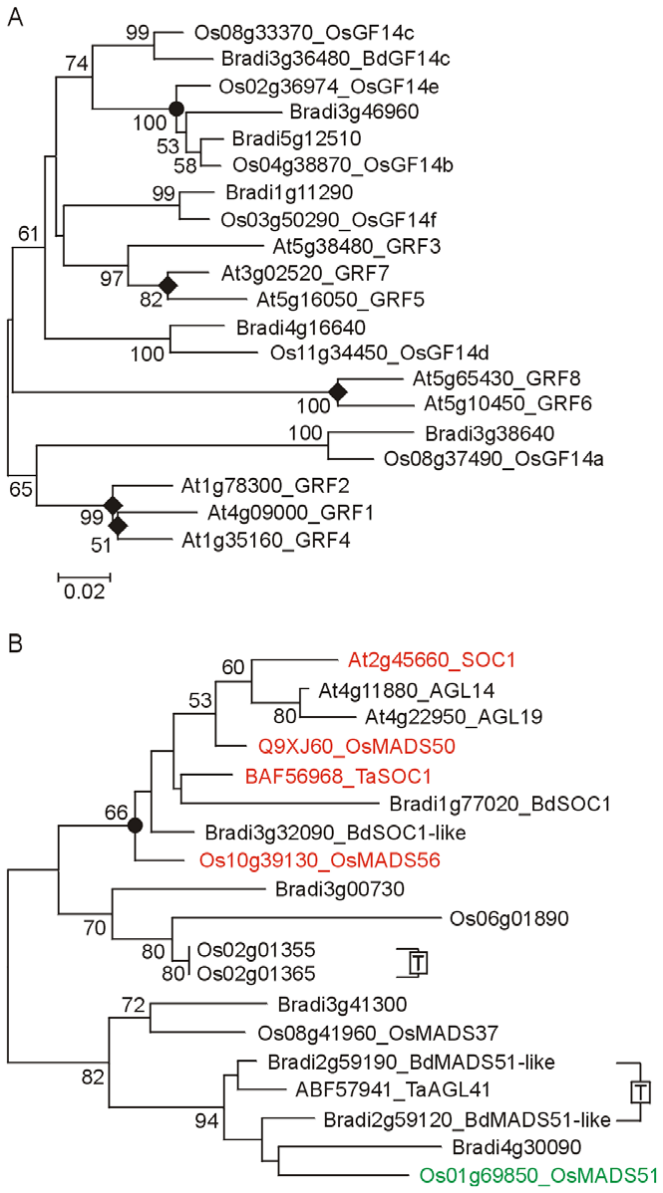
Phylogenetic relationship of 14-3-3 and SOC1 proteins. A) NJ tree of 14-3-3 and related proteins (whole protein alignment; Dataset S15). Only the 14-3-3 clade selected from Figure S6 is shown, this includes rice GF14c and Arabidopsis GRF5 (GF14υ) but excludes GRF9 (GF14μ) [80]. B) NJ tree of SUPRESSOR of CONSTANS 1 and related MADS-box proteins (whole protein alignment; Dataset S16). The SOC1 clade, including the MADS51 group, selected from Figure S7 is shown including the OsMADS50 (Q9XJ60) protein which spans Os03g03070 and Os03g03100.

FT is also regulated at the protein level by interaction with 14-3-3 proteins (PF00244) that typically modulate the activity of phosphorylation-dependent protein–protein interactions [78]. In rice, the GF14c Os08g0430500 protein (Os08g33370) acts as an inhibitor of flowering by binding to the Hd3a protein and thereby preventing activation of its targets [79]. GF14c falls in a clade containing six closely related rice proteins (Figure 9A); putative orthologs are present in Brachypodium suggesting that this type of control also exists in the temperate grasses. In Arabidopsis, 14-3-3 proteins are also implicated in the regulation of CO activity [80].

#### 2.3 SUPPRESSOR OF OVEREXPRESSION OF CO 1 (SOC1)

In Arabidopsis, FT upregulates the MADS-box transcription factor *SOC1* (*AGL20*) which is also controlled by the gibberellic acid pathway (not investigated in this paper) and repressed by FLOWERING LOCUS C (FLC) (Figure 1A). *SOC1* is also controlled post-transcriptionally by EARLY FLOWERING 9 (ELF9) (Figure 1 [81]). *SOC1*-like genes have been reported from rice and wheat but although the wheat gene can partially complement an Arabidopsis *soc1* mutation, its expression was not found to be regulated by photoperiod or vernalization [82]. However, two paralogous rice genes (*OsMADS50* and *56*) have been shown to be involved in flowering in long days by regulating *Ehd1* via antagonistic regulation of *LEC2 and FUSCA3 Like 1* (*LFL1*) (Figure 1 [83], [84]). Brachypodium orthologues of *OsMADS50* and *56* were found from the phylogenetic analysis but their relationship to Arabidopsis is complicated by the observation that the grass genes also resemble other Arabidopsis genes (*AGL14*, *19*, *42*, *71* and *72*; Figure 9B).

#### 2.4 LEAFY (LFY)

*RFL* (Os04g51000), the rice homologue of Arabidopsis *LFY*, has been shown to have a dual role. *RFL* acts as a regulator of plant architecture through its effects on apical and axillary meristems throughout the growth of the rice plant, but it appears to have a different expression profile compared to other grasses [85]. *RFL* affects flowering time and acts upstream of *OsMADS50* (*OsSOC1*) and *RFT1* to promote flowering [85]. This contrasts with Arabidopsis where *LFY* functions downstream of *SOC1* (Figure 1). The protein is well conserved and a Brachypodium homologue was found (Bradi5g20340) so differences in function may reflect changes in expression profile.

### 3. Vernalization pathways

In contrast to photoperiod, the control of flowering by exposure to extended periods of cold (vernalization) is generally thought to have evolved independently in Arabidopsis and grasses (reviewed by [11], [12], [16], [17], [18], [19]). However, in both cases there is repression of *FT* expression (Figure 1) and there are common components of epigenetic regulation [86]. Three genes determining vernalization requirement (*VERNALIZATION-1* (*VRN-1*), *VRN-2* and *VRN-3*) have been identified in wheat and barley using mutations that distinguish “winter” types (requiring vernalization) from “spring” types (no vernalization requirement). (N.B. the cereal *VRN* genes are unrelated to the *VRN* genes of Arabidopsis).

#### 3.1 VERNALIZATION-1 (VRN-1)

The grass *VRN-1* gene is related to the *FRUITFUL* (*FUL*)*/APETALA1* (*AP1*) subgroup of the MADS-box transcription factors in Arabidopsis [87], [88]. *AP1* is expressed in the shoot apex but the role of the grass *VRN-1* is more complex as it is expressed in vegetative tissues (leaf) as well as in the apex and may have different roles in these tissues [88].

**Figure 10:**
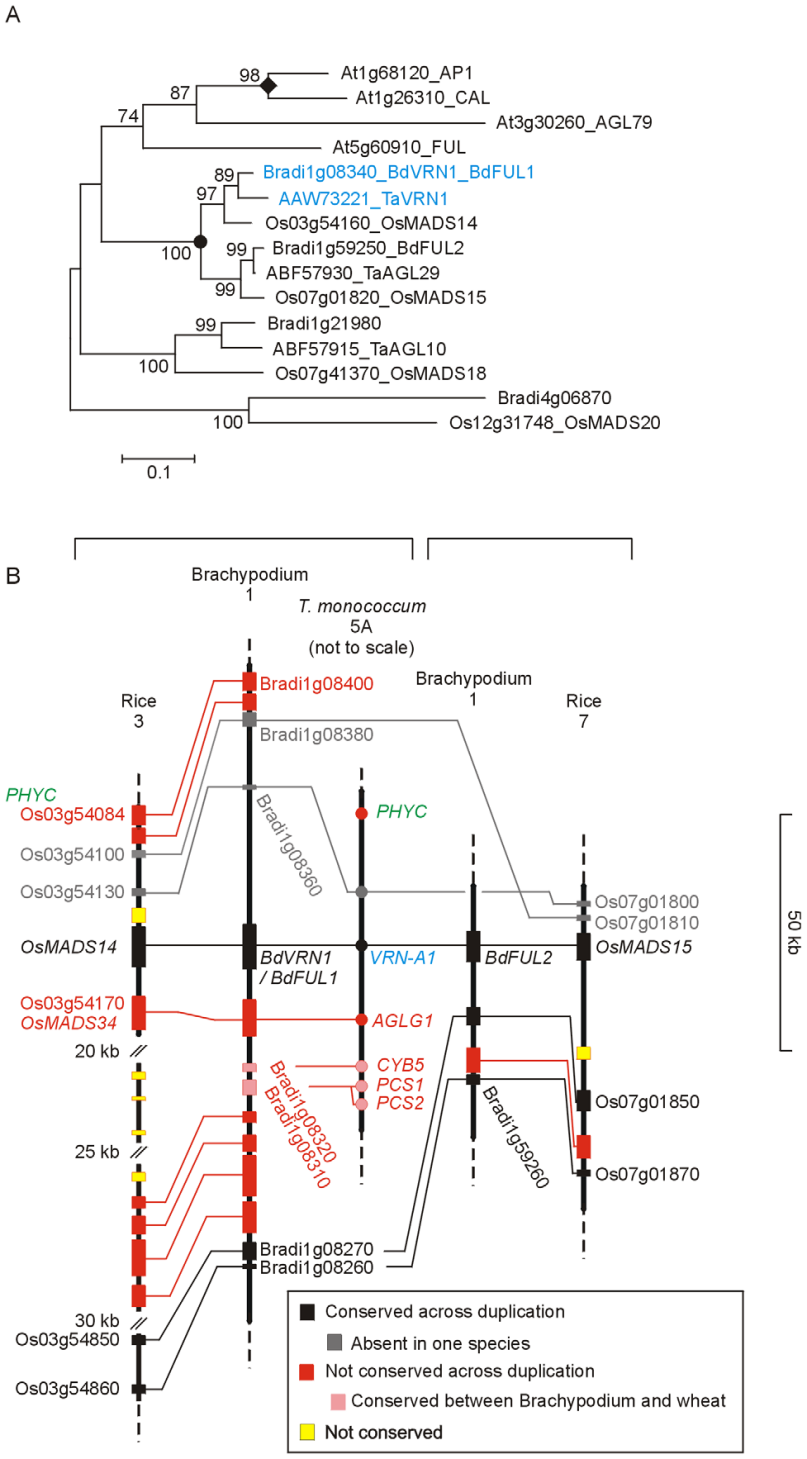
Phylogenetic relationship of FUL/AP1/VRN1 type MADS-box proteins and physical maps of the *FUL1* and *FUL2* regions. A) NJ tree of VRN1 and related proteins (whole protein alignment; Dataset S17). The FUL/AP1 clade selected from Figure S7 is shown. B) Structure of the segmental duplication containing the *FUL1* (*VRN1*; *OsMADS14*) and *FUL2* (*OsMADS15*) genes of the grasses.

In contrast to the Arabidopsis major flowering repressor *FLC* (section 5), *VRN-1* increases in expression during vernalization as plants move towards flowering competence [12], [18], [19], [87]. In cereals, mutations in the promoter or first intron of *VRN-1* create “spring” alleles that do not require vernalization, suggesting that they remove regulatory domains normally required for repressing gene expression (summarized in [16], [18]). In polyploid wheats, the presence of a single spring allele is sufficient to allow flowering without vernalization. Plants of diploid wheat (*T. monococcum*) which are homozygous for a deletion of *VRN-1* cannot flower, suggesting that *VRN-1* is essential for the transition from vegetative to reproductive growth [89], although it is not clear whether additional genes are also deleted. A *VRN-1* homologue is present in Brachypodium (*BdVRN1* or *BdFUL1*) in a colinear position with respect to rice (*OsMADS14*) and wheat (*Vrn-1*) genes (Figure 10B).

The *FUL*/*AP1* clade includes four genes in the grasses (Figure 10A). The *VRN-1*/*FUL1*/*OsMADS14* gene is a paralogue of the *FUL2*/*OsMADS15* gene arising from a segmental duplication (Figure 10B [88]). Both genes have been identified as controllers of flowering in rice but only *VRN-1* is currently known to have a role in temperate grasses. There is likely to be some diverged function for the two genes as they have differences in expression pattern [88]. The third gene and fourth rice genes, *OsMADS18* and *OsMADS20* both had temperate grass orthologues.

#### 3.2 VERNALIZATION-2 (VRN-2)

**Figure 11:**
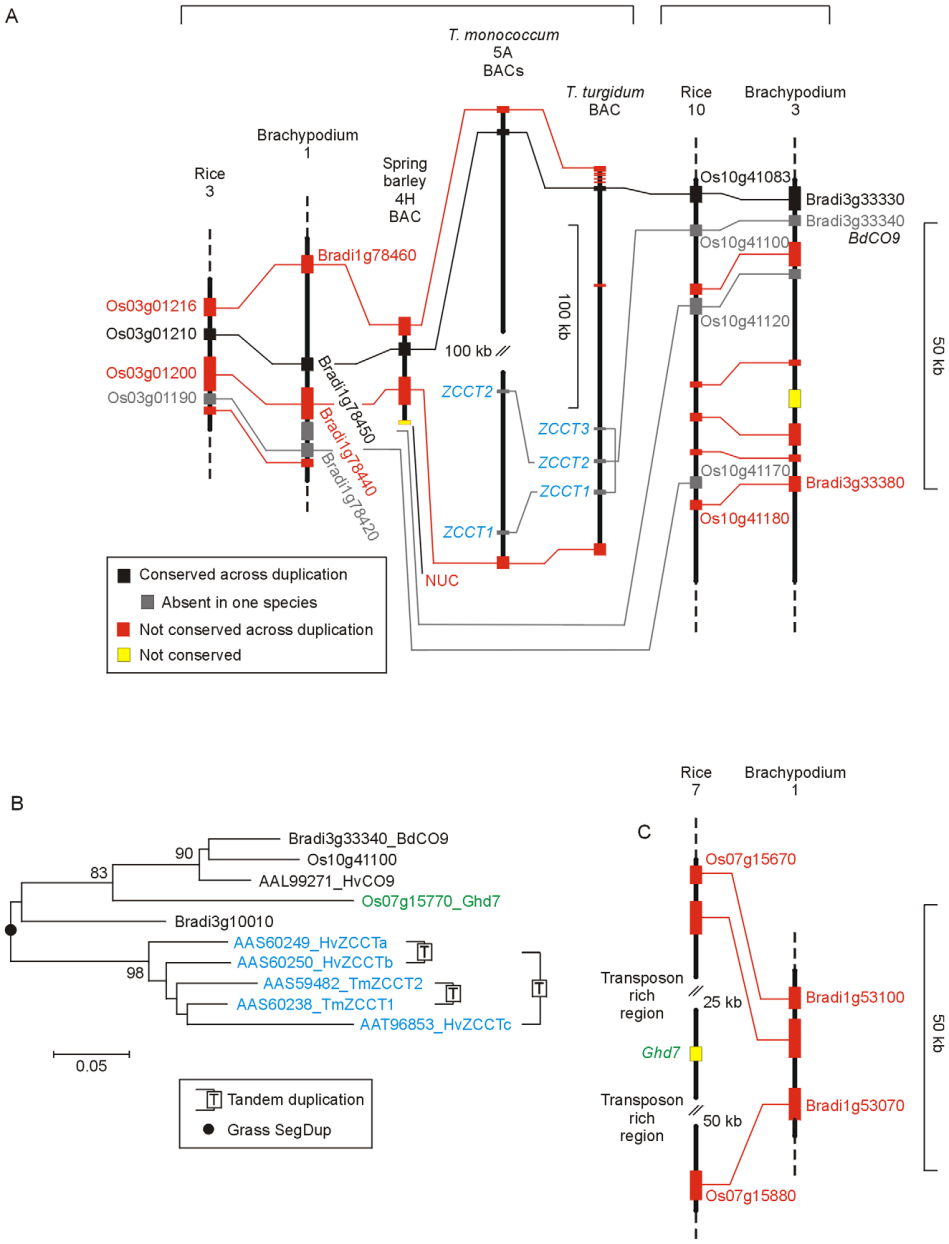
Phylogenetic relationship of VRN2/ZCCT proteins and physical maps of the *VRN2* region. A) Structure of the segmental duplication containing the *ZCCT* genes. B) NJ tree of ZCCT and related proteins (whole protein alignment; Dataset S18). C) Colinear regions of rice chromosome 7 and Brachypodium chromosome 1 showing the absence of a *Ghd7* homologue from Brachypodium.

*VRN-2* is a dominant repressor of flowering that behaves genetically in a similar way to the Arabidopsis *FLC* gene [90]. In contrast to *FLC* (encoding a MADS-box protein), *VRN-2* encodes a zinc-finger and CCT domain protein also called ZCCT1 [90]. *VRN-2* is a member of the group IV subfamily of *CO*-like CCT domain genes [51]. This subfamily is not found in Arabidopsis [51], [90]. Cereals have two or three *ZCCT* genes arranged in tandem (Figure 11A) but experimental evidence suggest that *ZCCT1* plays the important role in vernalization [90]. In contrast to *FLC*, *VRN-2* is controlled by day length as well as temperature and is only expressed in long days [91], [92]. The group IV subfamily of CCT genes also contains a long- day repressor in rice (*Ghd7* [93]) suggesting that long-day repression might be an ancestral feature and that regulation by cold was recruited to the cereal flowering pathway at a later date.

Brachypodium Bd21 resembled rice in having no *VRN-2* gene. Flanking genes from sequenced wheat BAC clones were present in collinear order suggesting that the gene is deleted (Figure 11A). This could mean that Brachypodium does not use vernalization as a flowering control in the same way as temperate cereals. However, Brachypodium accessions vary in flowering characteristics and Bd21 is a rapid flowering form with no requirement for vernalization [94]. This suggests that Bd21 resembles the *vrn-2* mutant of spring barley in having a complete deletion of the gene and that a *VRN-2* orthologue will be present in Brachypodium accessions with a vernalization requirement. Genetic mapping and sequencing of additional accessions will show if this is the case.

Although no *VRN-2* gene was found, Brachypodium did have two group IV CCT genes (Figure 11B). Bradi3g33340 is orthologous to *HvCO9*. A role for *CO9* in flowering has not been shown but is plausible as this gene is a paralogue of *VRN-2* arising from segmental duplication (Figure 11A). The absence of *VRN-2* in Bd21 and the segmental relationship to *HvCO9* has also been described by Cockram et al. [95]. The second Brachypodium gene (Bradi3g10010) was intermediate between *HvCO9* and *VRN-2* in the phylogenetic tree (Figure 11B) and had a diverged first exon compared to the latter. Brachypodium had no *Gdh7* homologue although genes flanking *Ghd7* in rice were well conserved and in collinear order (Figure 11C).

#### 3.3 VERNALIZATION-3 (VRN-3)

*VRN-3* is the *FT1* gene (section 2.1). The spring mutation in wheat is a transposon insertion in the promoter but the basis in barley is less clear. However, in both cases it is likely that there is loss of a regulatory region that normally allows repression by the vernalization pathway [96].

### 4. Meristem identity genes

**Figure 12:**
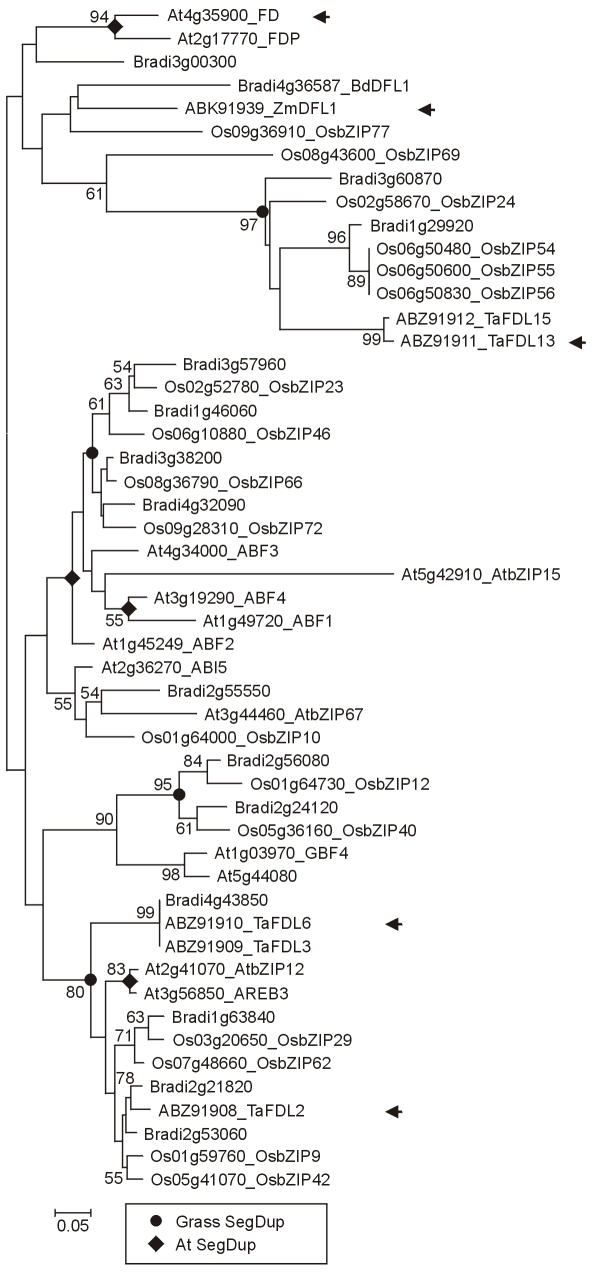
Phylogenetic relationship of FD and related bZIP proteins. NJ tree of FD and related basic region leucine zipper domain proteins (BRLZ (SM00338) domain alignment; Dataset S19). The bZIP clade was identified with an online interrogatory tree using known Arabidopsis flowering-time genes as query sequences [98]. Black arrows show genes with roles in flowering as described in [66], [67], [92], [94].

A key stage in flowering is the transition of the apical meristem from the vegetative phase (the production of leaves) to the reproductive phase (the production of flowers). In Arabidopsis the activation of *LFY*, *FUL* and *AP1* are important in this change (Figure 1). A protein complex containing FT and the basic leucine zipper (bZIP) domain protein FD activates *AP1* expression [68], [69] and this is likely to be conserved in other species including temperate grasses [97]. The bZIP clade (Figure 12) was identified using an online interrogatory tree [98] with Arabidopsis FD as the query sequence. This clade has several gene duplication events, complicating the interpretation of relationships.

*Delayed flowering1* (*DFL1*) in maize encodes a bZIP protein that acts downstream of *ID1* and mediates floral inductive signals at the shoot apex [99]. DFL1 is closely related to Arabidopsis FD in the phylogenetic tree; putative orthologues of DFL1 are present in Brachypodium and rice (Figure 12).

Five FD-like proteins have been identified in wheat of which TaFDL2 has been identified as a functional homologue of Arabidopsis FD because it interacts with both *TaFT* and the promoter of the wheat meristem identity gene *VRN-1* and is expressed in vegetative and reproductive apices [97]. In contrast to DFL1, TaFDL2 is not among the temperate grass proteins closely related to FD (Figure 12). These results suggest temperate grass genes more closely related to FD and DFL1 would be of interest for study.

*VRN-1* and its paralogue *FUL2* (section 3.1) are likely to have roles in meristem identity [88]. In Arabidopsis, the *LFY*, *FUL* and *AP1* genes are activated by *SQUAMOSA PROMOTER BINDING PROTEIN-LIKE 3* (*SPL3*) (Figure 1) which is regulated by *FT* and microRNA156 [100]. This control might also apply to *VRN-1* and/or *FUL2* in grasses. However, *SPL3* is a member of a large gene family in Arabidopsis and this was also the case in rice and Brachypodium, making identification of likely orthologues difficult (Figure S8).

### 5. The Arabidopsis vernalization and autonomous pathways

#### 5.1 FLOWERING LOCUC C (FLC)

**Figure 13:**
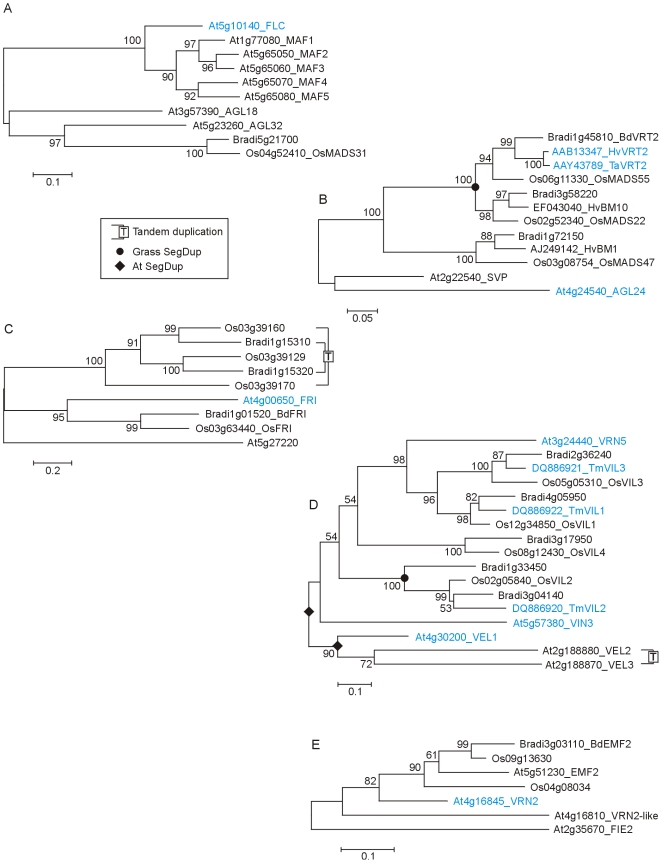
Phylogenetic relationships of FLC, SVP, FRI, VRN5 and polycomb-like proteins. A) NJ tree of FLOWERING LOCUS C and related MADS-box proteins (whole protein alignment; Dataset S20). Only the FLC clade selected from Figure S7 is shown. B) NJ tree of SHORT VEGETATIVE PHASE and related MADS-box proteins (whole protein alignment; Dataset S21). Only the SVP clade selected from Figure S7 is shown. C) NJ tree of FRIGIDA and related proteins (PF07899 domain alignment; Dataset S22). For Arabidopsis a functional FRI sequence (H51) was used. FRI and related clade selected from Figure S9. D) NJ tree of VERNALIZATION5 and related PHD finger proteins (whole protein alignment; Dataset S23). E) NJ tree of Arabidopsis VERNALIZATION2 and related polycomb group proteins (whole protein alignment; Dataset S24).

In Arabidopsis, the major flowering repressor in the vernalization pathway is the MADS-box gene *FLC* which represses *FT* and *FD* (Figure 1A; reviewed by [5]). Arabidopsis also has five *MADS AFFECTING FLOWERING* (*MAF*) genes which are homologous to *FLC* (Figure 13A). Vernalization represses *MAF1* (*FLM*), *MAF2* and *MAF3* but induces *MAF5* and does not affect *MAF4* [1], [101]. *MAF1* is a flowering repressor and *MAF2* functions in preventing short cold periods from inducing the vernalization response [1]. FLC and the MAFs formed a MADS-box subgroup lacking rice or Brachypodium members (Figure 13A), consistent with previous findings in the grasses (reviewed by [11], [12], [16], [17], [18], [19]). The *FLC*/*MAF* group of MADS-box genes is therefore likely to have evolved after the dicot/monocot divergence.

#### 5.2 AGAMOUS-LIKE 19 (AGL19)

In Arabidopsis, *AGL19* (related to *SOC1*) is regulated by vernalization (Figure 1A) but in contrast to *FLC* it is upregulated and acts as a floral promoter (reviewed by [1]). Rice and Brachypodium genes related to *SOC1* and *AGL19* were found and are described in section 2.3 (Figure 9B).

#### 5.3 SHORT VEGETATIVE PHASE (SVP) and AGAMOUS-LIKE 24 (AGL24)

In Arabidopsis, the MADS-box transcription factor SVP has a role in repressing *FT*, *TSF* and *SOC1* (Figure 1 [102]) and is likely to do this by interaction with FLC [103]. *SVP* also has a role in ambient temperature regulation [104]. SVP protein level is affected in *lhy*/*cca1* double mutants, suggesting that the clock also influences flowering by an additional route (not shown in Figure 1 [105]) which is also thought to involve *ELF3* [106]. *AGL24* is upregulated by vernalization and acts as a flowering promoter (reviewed by [1]).

*SVP*/*AGL24* like genes were found in rice and Brachypodium (Figure 13B) and have also been described in barley and wheat. In view of the lack of an *FLC* homologue in grasses the potential for *SVP*/*AGL24*-like genes to be involved in vernalization in grasses needs to be considered. Wheat *VEGETATIVE TO REPRODUCTIVE TRANSITION 2* (*TaVRT2*) is a member of this group that has been reported to be down regulated by vernalization and to interact with VRN2 protein to repress *VRN1* [107], [108]. However, other researchers disagree. Trevaskis et al. [109] did not find *VRT2* to be downregulated by vernalization and their studies of transgenic barley showed *SVP*-like genes to have roles in meristem development. *VRT2* is therefore shown in two possible positions in Figure 1C.

#### 5.4 FRIGIDA (FRI)

FRI, FRI-like (FRL1, FRL2), FRIGIDA ESSENTIAL 1 (FES1) and SUPPRESSOR OF FRIGIDA4 (SUF4) act to establish *FLC* expression (Figure 1A [110]). Genes with the closest homology to *FRI* in rice and Brachypodium were Os03g63440 and Bradi1g01520, respectively (Figure 13C). However, the sequences are considerably diverged from the Arabidopsis protein and it is unclear whether they are functionally related. For example, Arabidopsis FRI has two predicted coiled-coil domains [111] but neither was found in the rice or Brachypodium proteins. The relationships of the *FRI*-like genes are therefore ambiguous and functional assays of the rice and Brachypodium genes are needed to assess their function.

#### 5.5 Autonomous pathway genes

In Arabidopsis, *FLC* is downregulated by genes of the autonomous pathway (Figure 1A). In contrast to *FLC* and *FRI*, autonomous pathway genes are well conserved and are found in rice and Brachypodium (Table 1). FCA and FY interact and *FCA* transcript levels are controlled by alternative splicing which is conserved in grasses [112], [113]. Genes of the autonomous pathway are not solely concerned with the regulation of *FLC* and have functions in RNA-mediated chromatin silencing that affect other developmental processes [114], [115], [116]. These are plausible functions in other species and may include roles in flowering. *FCA* has also been shown to affect the level of miRNA172, providing a link to the photoperiod pathway in Arabidopsis [56]. *FCA*, *FY*, *FLOWERING LOCUS D* (*FLD*), *FPA*, *FVE* and *LUMINIDEPENDENS* (*LD*) were represented by orthologous single copy genes in rice and Brachypodium while *FLK* had two orthologues in rice and in Brachypodium (Table S1).

#### 5.6 Other genes repressing FLC

In Arabidopsis, *FLC* is repressed by vernalization in two phases, first by a repression of expression by cold and secondly by a stable maintenance of repression that persists after the plant returns to warm temperatures [5], [114]. These processes overlap and several genes function in both. The initial downregulation is thought to involve *VERNALIZATION INSENSITIVE 3* (*VIN3*) and *VERNALIZATION 5* (*VRN5*) which are related PHD-FNIII-VID (PHD) domain genes. Two additional members of this family, *VERNALIZATION5/VIN3-LIKE* (*VEL1*) and *VEL2* are also found in Arabidopsis. Four PHD genes from rice and three from *T. monococcum* were described previously, with the *T. monococcum* genes shown to be upregulated by vernalization [117]. Five genes were found in Brachypodium, four corresponding to the known rice genes with the fifth being related to *OsVIL2* (Figure 13D). As previously found [117], the grass genes are most closely related to *VRN5* and could play roles in gene regulation through chromatin changes. There were no genes highly homologous to *VIN3*, *VEL1*, *VEL2* or *VEL3*.

Stable repression of *FLC* involves a protein complex containing VRN5, VIN3, VEL1 and polycomb repressive complex 2 (PRC2) components VERNALIZATION 2 (VRN2; polycomb group protein), SWINGER (SWN; SET domain protein), FERTILIZATION INDEPENDENT ENDOSPERM 1 (FIE1; polycomb group) and MULTICOPY SUPRESSOR OF IRA1 (MSI1; WD repeat domain proteins) [118], [119]. Arabidopsis VERNALIZATION 1 (VRN1; a class VI REM type B3 domain protein [120]) was not found in rice or Brachypodium. Genes related to the PRC2 components were found in grasses and may have roles in gene regulation that involve changes to chromatin structure. This could include vernalization which, as in Arabidopsis, has an epigenetic “memory”. However, other flowering processes may also be regulated since *FT* has also been shown to have PRC2 control [121].

No convincing homologue of Arabidopsis *VRN2* was found in rice. Two genes of the polycomb type were found (Os09g13630 and Os04g08034) but they were more closely related to Arabidopsis *EMBRYONIC FLOWER 2* (*EMF2*). One gene was found in Brachypodium (Figure 13E). This was also *EMF2* like and was orthologous to Os09g13630. A second Brachypodium gene may be present on chromosome 4 but is not currently annotated.

Two SET domain genes *SWINGER* (*SWN*) and *CURLY LEAF* (*CLF*) have a role in vernalization in Arabidopsis; putative orthologs were found in both rice and Brachypodium (Table S1). Two WD repeat genes resembling *AtFIE1* were found in rice, positioned closely on chromosome 8 and separated only by a putative actin gene. Four possible genes were found in Brachypodium of which Bradi3g14520 was the closest relative. These results suggest that grasses contain chromatin regulatory genes related to PRC2 complexes, as might be expected, but lack convincing orthologues of key *FLC* regulators such as *VRN2*.

### 6. Ambient temperature pathways

In addition to control by vernalization, flowering in Arabidopsis is strongly affected by changes in ambient temperature. For example, a change in temperature from 23°C to 27°C (thermal induction) in short days promotes flowering as effectively as changing from short to long days [122]. Thermal induction is dependent on *FT* and *FD* but does not depend on *CRY1*, *CRY2*, *CO*, *GI* or *SOC1*, showing that *FT* is activated independently of the photoperiod pathway. Thermal induction is suppressed by FLC and by mutations of *FCA*, *FVE* and *FLD* that increase *FLC* expression [122].

Recent work in Arabidopsis shows that an important component of temperature response is the presence of histone H2A in place of H2A.Z in nucleosomes at higher temperatures (17°C vs. 27°C [123]). H2A.Z was depleted from the *FT* promoter at higher temperature, explaining the photoperiod independent induction of expression. ACTIN-RELATED PROTEIN 6 (APR6; Table 1) is part of the SWR1 complex that incorporates H2A.Z in nucleosomes. The *arp6* mutant cannot incorporate H2A.Z, constitutively behaves as if at a higher temperature and is early flowering, especially in short days, consistent with higher *FT* expression [123]. SWR1 is also required for *FLC* expression suggesting that nucleosome changes may affect multiple flowering-time genes. Cereals show a degree-day response and it would be of interest to quantify effect of temperature in relation to *FT* expression. Also, given the lack of an *FLC* equivalent and the day length dependent expression of the flowering repressor *VRN2* (ZCCT; section 3.2) it would be interesting to compare cereal temperature responses in short and long days.

## Discussion

We analyzed the genome of *Brachypodium distachyon* to identify homologues of genes likely to be involved in the control of flowering time. As well as using flowering-time genes, we used flanking genes from rice or other cereals, where available, to clarify the relationships of putative orthologues. The use of flanking genes or “genomic context” was especially useful for understanding cases where a gene was absent from Brachypodium. A prominent example of this was the absence of the *VRN2* gene which acts as a flowering repressor in the cereal vernalization pathway.

### The FT hub

The core photoperiod pathway (circadian clock-GI-CO-FT) defined in Arabidopsis is well conserved as are components of the autonomous pathway. However, there is good evidence that additional components and regulatory pathways have been added during the evolution of different lineages and a common feature of these, illustrated in Figure 1, is that they provide additional promoting or repressing controls of the floral pathway integrator *FT*. As FT is a key signalling molecule moving from the leaves to the apical meristem (reviewed by [9]) this suggests that adjustment of FT levels provides a convenient evolutionary solution when alteration of flowering time is needed. However, care must be taken in this interpretation because *FT* is studied in many experiments and other significant hubs might emerge as additional genes, and other species, are studied in more depth.

### Segmental duplications

The number of genes in a family is affected by the segmental duplications that are well characterized in rice and Brachypodium [124], [125]. The overall structure of the duplications we examined is well conserved, consistent with the idea that duplication occurred in an ancestral grass genome and assumed much of the modern structure before the divergence of the rice, Brachypodium and temperate grass lineages. Segmental duplication accounts for the presence of two grass genes resembling Arabidopsis *PRR7* (Figure 3), the presence of two *CO*-like genes (*CO1*, *CO2*) compared to one in rice (*Hd1*) (Figure 5) and the presence of two *FUL*/*AP1* like genes (*VRN1* and *FUL2*, Figure 10). Segmental duplication also shows the relationship between the grass *VRN2* (*ZCCT*) genes and *CO9* (Figure 11). Tandem duplications explain other differences such as the presence of one *FT* like gene in Brachypodium, barley and wheat (*FT1*) compared to two in rice (*Hd3a* and *RFT1*).

A significant feature of the segmental duplications is that multiple alleles conferring natural variation in flowering time in cereals are associated with only one of the genes in each pair. Variation in photoperiod response is provided by the *PRR37* gene in barley (*Ppd-H1*) and wheat (*Ppd-A1*, *-B1* and *–D1*, chromosomes 2A, 2B and 2D) but no such effect is known for the *PRR73* gene. Variation in vernalization requirement is provided by the *VRN-1* gene in barley and diploid, tetraploid and hexaploid wheat but no such effect is known for the *FUL2* gene. Variation in vernalization requirement is also provided by the *ZCCT* gene in barley (*Vrn-H2*) and diploid and tetraploid wheat (*Vrn-A2* and *Vrn-B2*) but no such effect is known for its counterpart *CO9*. This suggests that the gene pairs are diverged in function and that only one member can be mutated to provide useful variation, at least in an agricultural context. It would be interesting to explore this in wild grasses and to assess the function of the second member of each pair experimentally. Brachypodium would provide an excellent vehicle for this.

### Clear versus fuzzy homology

Some genes such as *CO* and *GI* had convincing orthologues in all species, but other genes were harder to interpret. For example, the relationship between monocot and dicot members of the *PRR*, *CDF1*, *TOE1*, *TEM* and *HAP* genes is complex and is likely to reflect different patterns of expansion and contraction in these families since the divergence of Arabidopsis, rice and Brachypodium. However, even within these families, rice and Brachypodium genes were generally associated in pairs in the phylogenetic trees, suggesting that it will be possible to integrate knowledge of gene function between grasses if not between grasses and Arabidopsis.

### The evolution of novel pathways

There are clear examples of distinct genetic controls in different lineages. The vernalization pathways are a prominent example, with *FLC* encoding the main repressor of flowering in Arabidopsis and *VRN2* in cereals. The absence of *VRN2* from Brachypodium was an unexpected finding but this may reflect the choice of accession used for sequencing. Analysis of additional Brachypodium accessions for the presence of *VRN2* would be of great interest.

Other examples are *Vgt1* from maize and *Ehd1* and *Ghd7* from rice which were not found in Brachypodium, and *MADS51* and *ID1* which were found in rice and Brachypodium but not in Arabidopsis. These genes are members of families and there is therefore the risk that “fuzzy” homology may be missed. This problem would be exacerbated by incorrect gene prediction. We therefore checked these examples carefully, especially as homologues of positive and negative regulators of *Ehd7* and *Ghd7* were found. We reanalyzed relevant regions using reciprocal TBLASTN searches of nucleotide sequence but were unable to find homologous sequences and we conclude that these genes are genuinely absent. As with *VRN2*, it would be interesting to explore further accessions to see if these genes are consistently absent from particular species or if gain or loss reflects local adaptation.

Our analysis used known flowering-time genes from species other than Brachypodium. As Brachypodium transformation is now established [126] it will be possible to experimentally test conservation of gene function. Novel flowering-time genes may be identified in Brachypodium itself from mutation screens or from the analysis of different ecotypes. The latter also provide an excellent system to test if flowering time variation between wild ecotypes involves the same genes that have been selected in cereals during domestication.

## Materials and Methods

### Sources of sequences

The following datasets were used for profile Hidden Markov Models (HMMs) analysis and BLAST searches: Arabidopsis TAIR9 (http://www.arabidopsis.org, TAIR9_pep_20090619), Rice MSU Osa1 Release 6.1 (http://rice.plantbiology.msu.edu, all.pep – June 2009) and Brachypodium v1 (http://www.modelcrop.org, Bd21_v1_Protein[1].fa – May 2009). Updated annotations for Brachypodium are available in Brachypodium v1.2. The representative gene model was used; for most genes this is the ‘.1’ version. If the alternative gene model version (‘.2’, ‘.3’) is used this is indicated in the phylogenetic trees (supplement) or in Table S1.

Protein sequences for wheat, barley and maize flowering-time genes were obtained from UNIPROT (http://www.uniprot.org) or NCBI (http://www.ncbi.nlm.nih.gov) and Sorghum sequences were obtained from (http://genome.jgi-psf.org/Sorbi1).

### Homologue identification in rice and Brachypodium

Starting with Arabidopsis flowering-time genes (Table 1), BLASTP searches were carried out against rice protein sequences with an E-value threshold of 1e^−20^. The top ranked rice hit was used for BLASTP searches back into the Arabidopsis protein to confirm homology (reciprocal BLAST searching). Starting with both Arabidopsis and rice homologues, BLASTP and TBLASTN searches were carried out against the Brachypodium v1 protein predictions and genomic sequence, respectively. Putative orthologues were confirmed by reciprocal BLASTP searches of the Arabidopsis and rice protein sequence datasets and are summarized in Table S1. Genes that have been shown experimentally to have a role in flowering time in grasses were also included in the BLAST analyses. A ClustalW alignment (http://www.ebi.ac.uk/Tools/clustalw2) was performed for each gene set and the clustal score recorded as a measure of protein sequence identity (Table 1). For flowering-time genes that belong to large gene families where duplication and gene loss complicated the identification of homologues the following methods were used: 1) profile HMMs searches to identify all members of each family or subfamily followed by phylogenetic analysis, 2) genomic location. Specific strategies for finding homologues are discussed in the result section for the individual gene families.

### Database searches using profile HMMs

For the larger transcription factor (TF) families (AP2 (TOE), ERF-B3 (TEM), bZIP (FD), NF-YB-like (HAP), Dof zinc finger (CDF), C2H2 zinc finger (ID), MADS-box) TCP (CHE), bHLH (CIB1), genes for particular subgroups were identified from Arabidopsis, rice and Brachypodium protein sequences by searches with profile HMMs using the HMMER suite of programs [24]. The profile HMM for each family was made from an alignment of the conserved region (centred around the DNA binding domain), seeded by protein sequences that were already known to play a role in the flowering time response and other close homologues (see main text for references). For the MADS-box family, the profile HMM was made from an alignment of all members of this family present in Arabidopsis, excluding sequences that did not align correctly. The full alignment was used in the case of the ERF-B3, CDF and ID families.

For some TF families, there was a cut off in the list of hits from the HMMSEARCH program below which it was clear that sequences belonged to different subgroups of the gene family (ERF-B3, Dof). For other families (AP2, C2H2 zinc finger, bZIP, TCP and bHLH), the boundary of the relevant subgroup was found by adding the sequence hits as queries to an online interrogatory tree for each family [98]. It was necessary to identify and perform phylogenetic analysis on all members of the MADS box family to identify the members of each subgroup of interest in the MIKC-type MADS-box proteins. In the case of the NF-YB-like (HAP) family, an E-value cut off of 1.0 was applied to the HMMSEARCH results.

The above approach was also adopted to search for all members of the 14-3-3, FRIGIDA-like and SPL gene families. In these cases, an existing profile HMM from the pFAM website was used, PF00244, PF07899 and PF03110 respectively.

All sequence matches selected from the HMMSEARCH output were aligned using PRANK [25], except for the HAP family whose matches were aligned back to the HMM using the HMMALIGN program. The resulting alignments were used for phylogenetic analysis.

### Phylogenetic Analysis

The alignment for each gene family data set (see supplementary data) was used for distance-based phylogenetic analysis with the PHYLIP [127] or MEGA4.0 [128] software packages. With both packages, identical methods were used to calculate distance estimates (the Jones Taylor Thornton matrix (JTT) model of evolution) and to construct phylogenetic trees (Neighbor-Joining method). Each tree was rooted using the midpoint rooting method. To provide statistical support for each node, a consensus tree was generated from 100 (supplement) or 1000 bootstrap data sets.

From the larger trees shown as supplementary data, genes from particular subclades were taken and a second smaller tree generated for the main manuscript to illustrate the relationship of genes of interest and to include additional closely related genes from barley, wheat, maize or sorghum where relevant. Where appropriate, a longer alignment was used for the phylogenetic analysis (see supplementary data).

### Protein domains

Protein domains and secondary structure were analysed using PROSITE at the ExPASY facility (http://us.expasy.org/).

### Genome Duplication Data

Gene duplication data was obtained from three sources, the Plant Genome Duplication Database (PGDD - http://chibba.agtec.uga.edu/duplication/ [26], the Rice Genome MSU release 6.1 segmental duplications (http://rice.plantbiology.msu.edu/segmental_dup) and duplicated blocks in Arabidopsis [129]. Segmental duplications events were indicated in the phylogenetic tree diagrams for Arabidopsis and the grasses (rice and Brachypodium).

## Supporting Information

Table S1 Complete list of Arabidopsis, Rice, Brachypodium and Cereal flowering time related proteins. Extended version of Table 1 showing details of accession numbers, reciprocal blast hits and EST evidence.
(0.08 MB XLS)

Figure S1 The relationship between Arabidopsis CHE and closely related TCP proteins. The region of the alignment used to estimate the tree spanned the TCP domain. Although there was no significant bootstrap value leading to the clade that contained CHE, there was sequence conservation outside the DNA binding domain for all six proteins in the clade. The sequence in the C-terminal end showed that the Bradi3g60350 protein was mostly closely related to CHE and At5g23280 relative to the other proteins in the clade. This indicates that Brachypodium contains an orthologue of CHE that rice has lost.
(0.09 MB PPT)

Figure S2 The relationship between Arabidopsis TOE1 and other closely related proteins in the AP2 family. An alignment of both repeats that comprised the AP2 domain was used to estimate the tree.
(0.09 MB PPT)

Figure S3 The relationship between the monocot ID1 proteins and other ID domain proteins. The alignment for estimating the tree contained only the region of the four tandemly arranged zinc finger domains, excluding a small number of columns containing non-homologous amino acids. A neighbouring subgroup within a full C2H2 zinc finger family tree is also shown at the base of the tree, illustrating that the main subgroup shown is distinct from other proteins of this family. The monocot ID1 proteins within this subgroup form another distinct, internal subgroup.
(0.08 MB PPT)

Figure S4 The relationship between Arabidopsis CIB1 and other proteins in subgroup 12 of the bHLH family. The region of the alignment used to estimate the tree spanned the bHLH domain and adjacent regions that were also conserved in proteins belonging to this subgroup. Neither bootstrap analysis nor studying regions outside this region provided evidence for an orthologue of Arabidopsis CIB1 in Brachypodium.
(0.08 MB PPT)

Figure S5 The relationship between members of the NF-YB-like (HAP) family. A conserved region of eighty four amino acids was identified in an alignment of these proteins and used to estimate the tree. For this family, the alignment was created by aligning the sequences to a profile HMM of the conserved region.
(0.11 MB PPT)

Figure S6 The relationship between the rice GF14c protein and the rest of the 14-3-3 protein family. The full alignment was used to estimate the tree, except that the alignment ends were trimmed.
(0.07 MB PPT)

Figure S7 The relationship between flowering time genes that belong to the MADS-box family. The alignment was created by aligning all the sequences corresponding to this family to a profile HMM of the MADS-box domain. Nineteen poorly aligned proteins were removed from the data set before estimating the tree.
(0.17 MB PPT)

Figure S8 The relationship between SPL proteins. The region of the alignment used to estimate the tree corresponded to the pFAM profile HMM (PF03110) but excluded columns containing non-homologous amino acids.
(0.08 MB PPT)

Figure S9 The relationship between the FRIGIDA protein and related proteins. The region of the alignment used to estimate the tree corresponded to the pFAM profile HMM (PF07899) but excluded columns containing non-homologous amino acids.
(0.07 MB PPT)

Dataset S1 Fasta formatted protein sequences for HvPRR genes (Figure 3).
(0.00 MB TXT)

Dataset S2 Fasta formatted alignment of CKIIa proteins used for tree in Figure 2A.
(0.01 MB TXT)

Dataset S3 Fasta formatted alignment of ZTL proteins used for tree in Figure 2B.
(0.01 MB TXT)

Dataset S4 Fasta formatted alignment of ELF3 proteins used for tree in Figure 2C.
(0.01 MB TXT)

Dataset S5 Fasta formatted alignment of ELF4 proteins used for tree in Figure 2D.
(0.00 MB TXT)

Dataset S6 Fasta formatted alignment of PRR proteins used for tree in Figure 3A.
(0.00 MB TXT)

Dataset S7 Fasta formatted alignment of CDF proteins used for tree in Figure 4.
(0.04 MB TXT)

Dataset S8 Fasta formatted alignment of CO proteins used for tree in Figure 5A.
(0.03 MB TXT)

Dataset S9 Fasta formatted alignment of TOE proteins used for tree in Figure 6A.
(0.03 MB TXT)

Dataset S10 Fasta formatted alignment of TEM proteins used for tree in Figure 7A.
(0.01 MB TXT)

Dataset S11 Fasta formatted alignment of ID domain proteins used for tree in Figure 7C.
(0.00 MB TXT)

Dataset S12 Fasta formatted alignment of FT proteins used for tree in Figure 8A.
(0.01 MB TXT)

Dataset S13 Fasta formatted alignment of HAP3 proteins used for tree in Figure 8B.
(0.01 MB TXT)

Dataset S14 Fasta formatted alignment of HAP5 proteins used for tree in Figure 8C.
(0.01 MB TXT)

Dataset S15 Fasta formatted alignment of 14-3-3 proteins used for tree in Figure 9A.
(0.01 MB TXT)

Dataset S16 Fasta formatted alignment of SOC1 and MADS51 proteins used for tree in Figure 9B.
(0.02 MB TXT)

Dataset S17 Fasta formatted alignment of VRN1 proteins used for tree in Figure 10A.
(0.01 MB TXT)

Dataset S18 Fasta formatted alignment of VRN2/ZCCT proteins used for tree in Figure 11B.
(0.00 MB TXT)

Dataset S19 Fasta formatted alignment of FD proteins used for tree in Figure 12.
(0.01 MB TXT)

Dataset S20 Fasta formatted alignment of FLC proteins used for tree in Figure 13A.
(0.00 MB TXT)

Dataset S21 Fasta formatted alignment of SVP proteins used for tree in Figure 13B.
(0.00 MB TXT)

Dataset S22 Fasta formatted alignment of FRI proteins used for tree in Figure 13C.
(0.00 MB TXT)

Dataset S23 Fasta formatted alignment of VRN5 proteins used for tree in Figure 13D.
(0.02 MB TXT)

Dataset S24 Fasta formatted alignment of VRN2 proteins used for tree in Figure 13E.
(0.01 MB TXT)
