# Supplementary material for: Comparative Genomics of Flowering Time Pathways Using Brachypodium distachyon as a Model for the Temperate Grasses
Source: PLoS One. 2010 Apr 19;5(4):e10065. doi: 10.1371/journal.pone.0010065 (PMC2856676; doi:10.1371/journal.pone.0010065)
Supplement: Figure S1 — The relationship between Arabidopsis CHE and closely related TCP proteins. The region of the alignment used to estimate the tree spanned the TCP domain. Although there was no significant bootstrap value leading to the clade that contained CHE, there was sequence conservation outside the DNA binding domain for all six proteins in the clade. The sequence in the C-terminal end showed that the Bradi3g60350 protein was mostly closely related to CHE and At5g23280 relative to the other proteins in the clade. This indicates that Brachypodium contains an orthologue of CHE that rice has lost. (0.09 MB PPT) [file pone.0010065.s002.ppt]

## Slide 1
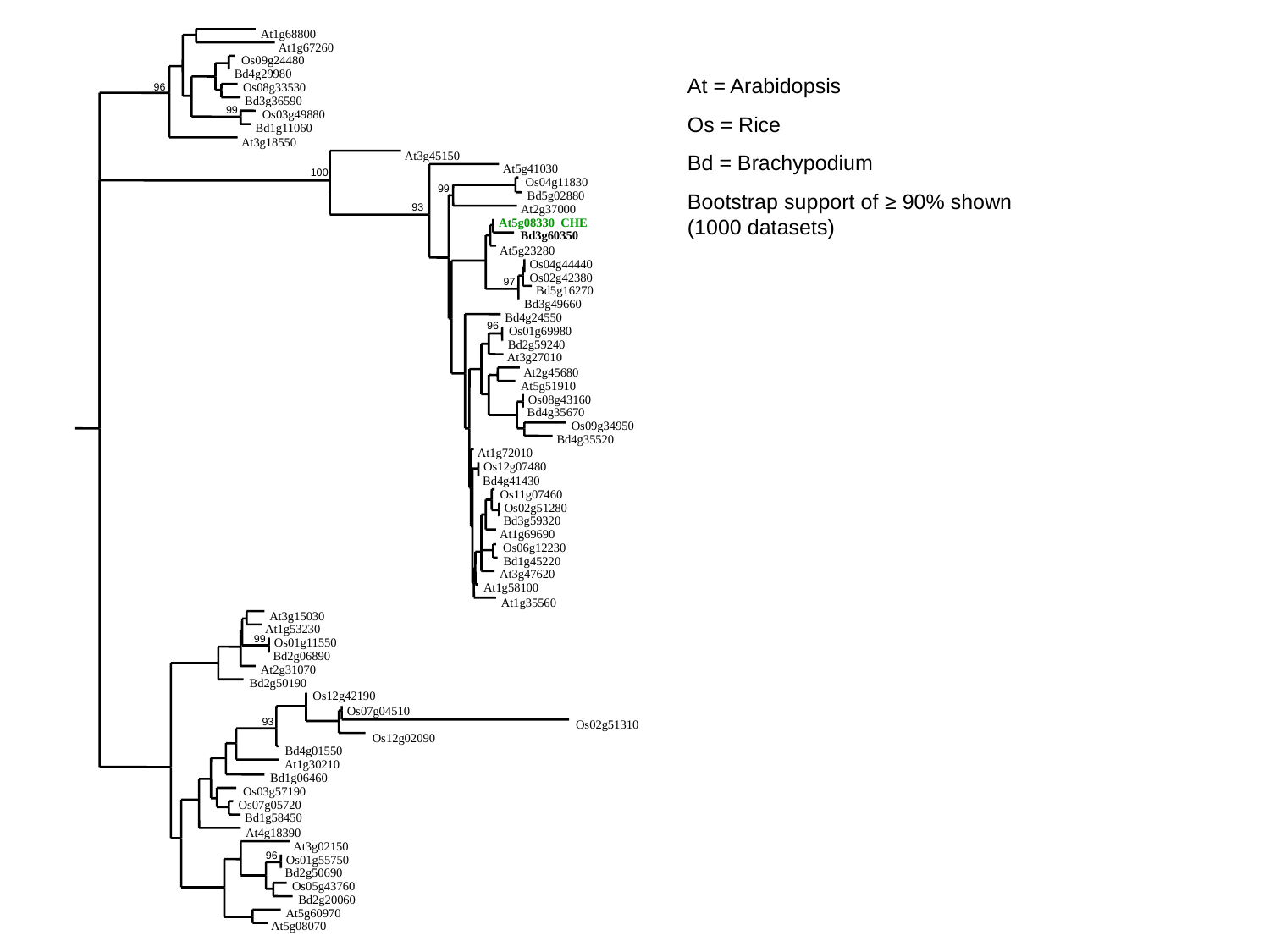

At1g68800
At1g67260
Os09g24480
Bd4g29980
Os08g33530
Bd3g36590
Os03g49880
Bd1g11060
At3g18550
At3g45150
At5g41030
Os04g11830
Bd5g02880
At2g37000
At5g08330_CHE
Bd3g60350
At5g23280
Os04g44440
Os02g42380
Bd5g16270
Bd3g49660
Bd4g24550
Os01g69980
Bd2g59240
At3g27010
At2g45680
At5g51910
Os08g43160
Bd4g35670
Os09g34950
Bd4g35520
At1g72010
Os12g07480
Bd4g41430
Os11g07460
Os02g51280
Bd3g59320
At1g69690
Os06g12230
Bd1g45220
At3g47620
At1g58100
At1g35560
At3g15030
At1g53230
Os01g11550
Bd2g06890
At2g31070
Bd2g50190
Os12g42190
Os07g04510
Os02g51310
Os12g02090
Bd4g01550
At1g30210
Bd1g06460
Os03g57190
Os07g05720
Bd1g58450
At4g18390
At3g02150
Os01g55750
Bd2g50690
Os05g43760
Bd2g20060
At5g60970
At5g08070
At = Arabidopsis
Os = Rice
Bd = Brachypodium
Bootstrap support of ≥ 90% shown
(1000 datasets)
96
99
100
99
93
97
96
99
93
96
