# Supplementary material for: Comparative Genomics of Flowering Time Pathways Using Brachypodium distachyon as a Model for the Temperate Grasses
Source: PLoS One. 2010 Apr 19;5(4):e10065. doi: 10.1371/journal.pone.0010065 (PMC2856676; doi:10.1371/journal.pone.0010065)
Supplement: Figure S2 — The relationship between Arabidopsis TOE1 and other closely related proteins in the AP2 family. An alignment of both repeats that comprised the AP2 domain was used to estimate the tree. (0.09 MB PPT) [file pone.0010065.s003.ppt]

## Slide 1
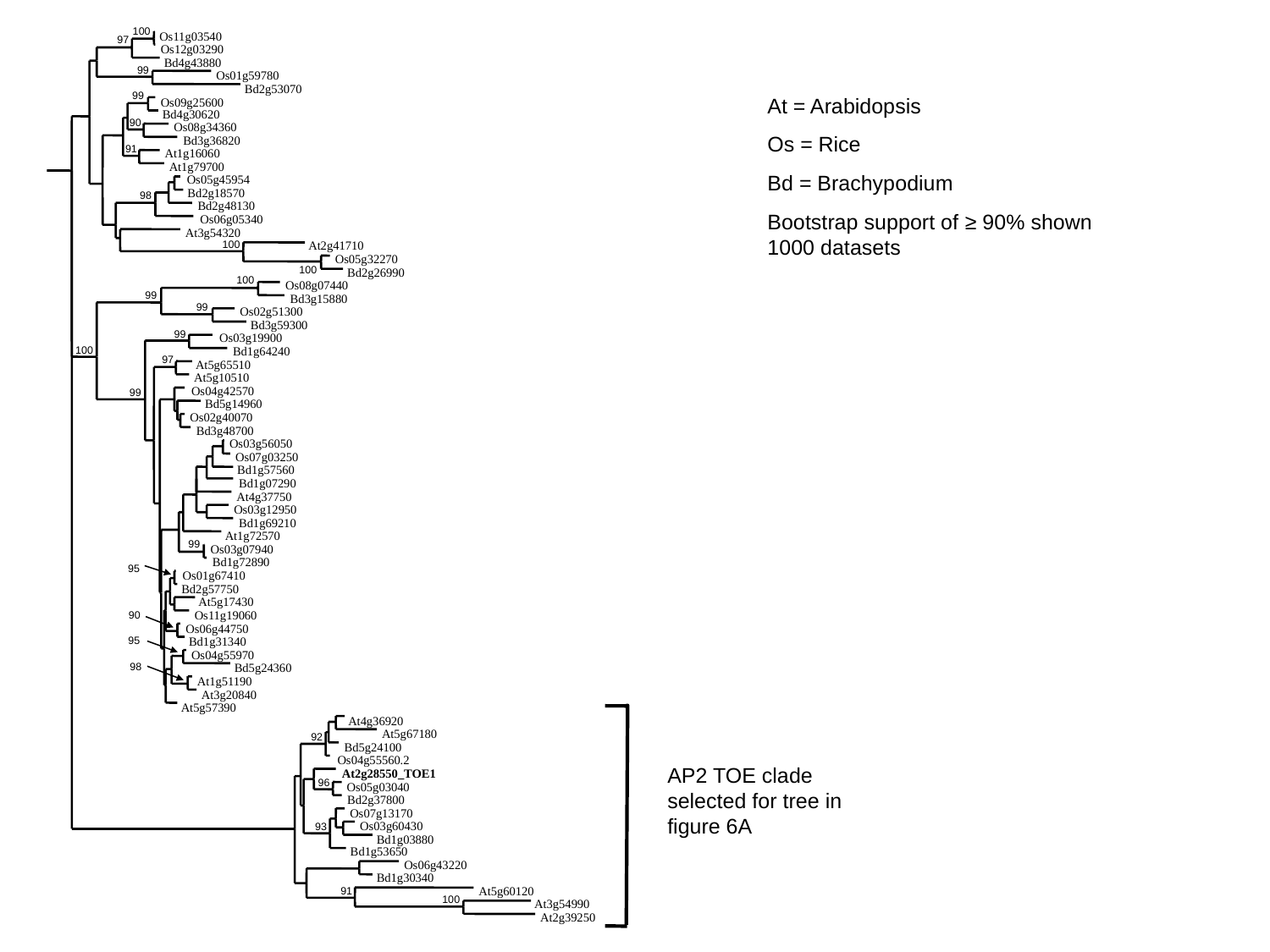

100
97
Os11g03540
Os12g03290
Bd4g43880
Os01g59780
Bd2g53070
Os09g25600
Bd4g30620
Os08g34360
Bd3g36820
At1g16060
At1g79700
Os05g45954
Bd2g18570
Bd2g48130
Os06g05340
At3g54320
At2g41710
Os05g32270
Bd2g26990
Os08g07440
Bd3g15880
Os02g51300
Bd3g59300
Os03g19900
Bd1g64240
At5g65510
At5g10510
Os04g42570
Bd5g14960
Os02g40070
Bd3g48700
Os03g56050
Os07g03250
Bd1g57560
Bd1g07290
At4g37750
Os03g12950
Bd1g69210
At1g72570
Os03g07940
Bd1g72890
Os01g67410
Bd2g57750
At5g17430
Os11g19060
Os06g44750
Bd1g31340
Os04g55970
Bd5g24360
At1g51190
At3g20840
At5g57390
At4g36920
At5g67180
Bd5g24100
Os04g55560.2
At2g28550_TOE1
Os05g03040
Bd2g37800
Os07g13170
Os03g60430
Bd1g03880
Bd1g53650
Os06g43220
Bd1g30340
At5g60120
At3g54990
At2g39250
99
99
At = Arabidopsis
Os = Rice
Bd = Brachypodium
Bootstrap support of ≥ 90% shown
1000 datasets
90
91
98
100
100
100
99
99
99
100
97
99
99
95
90
95
98
92
AP2 TOE clade selected for tree in figure 6A
96
93
91
100
