# Supplementary material for: Comparative Genomics of Flowering Time Pathways Using Brachypodium distachyon as a Model for the Temperate Grasses
Source: PLoS One. 2010 Apr 19;5(4):e10065. doi: 10.1371/journal.pone.0010065 (PMC2856676; doi:10.1371/journal.pone.0010065)
Supplement: Figure S3 — The relationship between the monocot ID1 proteins and other ID domain proteins. The alignment for estimating the tree contained only the region of the four tandemly arranged zinc finger domains, excluding a small number of columns containing non-homologous amino acids. A neighbouring subgroup within a full C2H2 zinc finger family tree is also shown at the base of the tree, illustrating that the main subgroup shown is distinct from other proteins of this family. The monocot ID1 proteins within this subgroup form another distinct, internal subgroup. (0.08 MB PPT) [file pone.0010065.s004.ppt]

## Slide 1
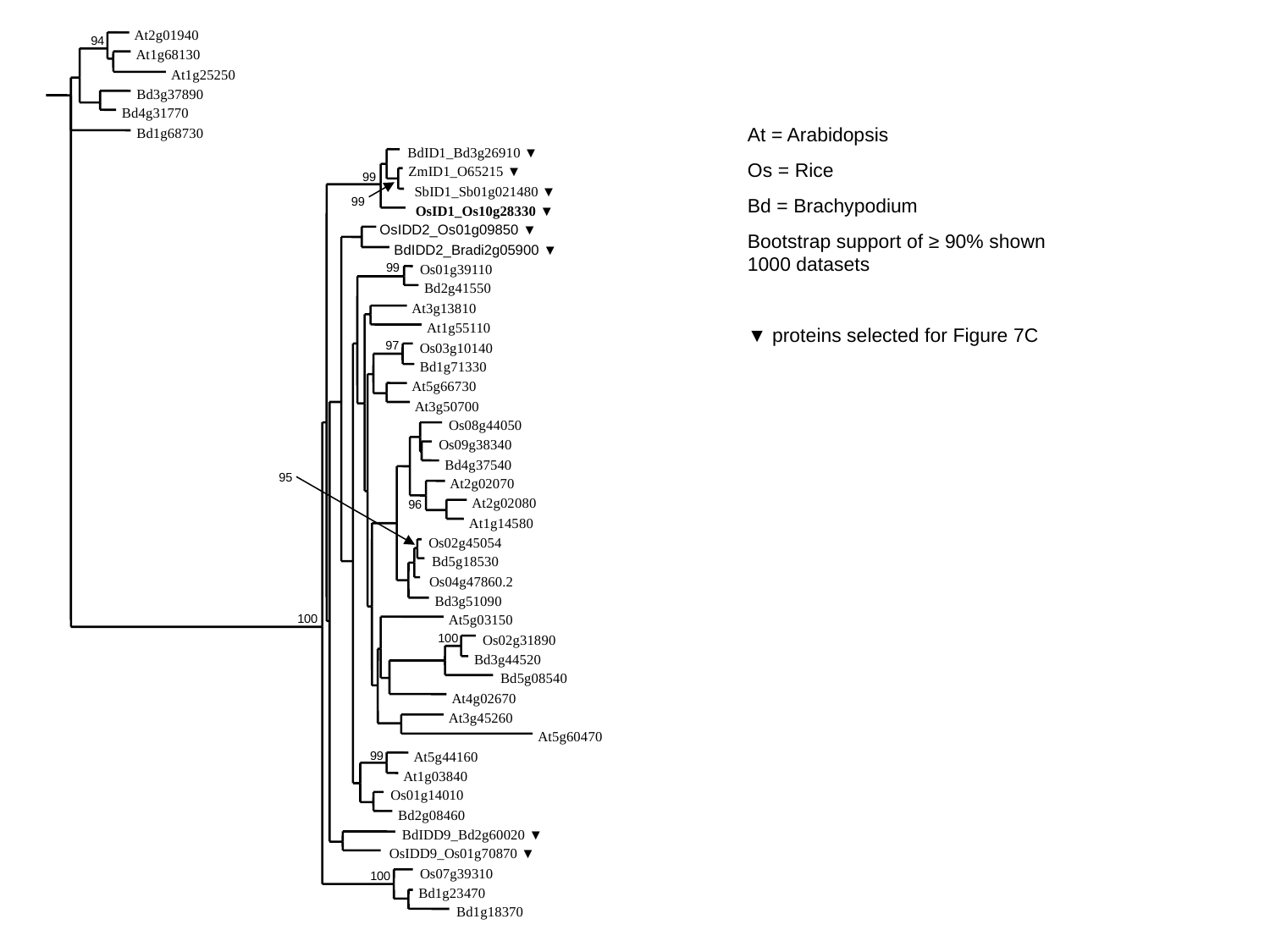

94
At2g01940
At1g68130
At1g25250
Bd3g37890
Bd4g31770
Bd1g68730
BdID1_Bd3g26910 ▼
ZmID1_O65215 ▼
SbID1_Sb01g021480 ▼
OsID1_Os10g28330 ▼
OsIDD2_Os01g09850 ▼
BdIDD2_Bradi2g05900 ▼
Os01g39110
Bd2g41550
At3g13810
At1g55110
Os03g10140
Bd1g71330
At5g66730
At3g50700
Os08g44050
Os09g38340
Bd4g37540
At2g02070
At2g02080
At1g14580
Os02g45054
Bd5g18530
Os04g47860.2
Bd3g51090
At5g03150
Os02g31890
Bd3g44520
Bd5g08540
At4g02670
At3g45260
At5g60470
At5g44160
At1g03840
Os01g14010
Bd2g08460
BdIDD9_Bd2g60020 ▼
OsIDD9_Os01g70870 ▼
Os07g39310
Bd1g23470
Bd1g18370
At = Arabidopsis
Os = Rice
Bd = Brachypodium
Bootstrap support of ≥ 90% shown
1000 datasets
▼ proteins selected for Figure 7C
99
99
99
97
95
96
100
100
99
100
