# Supplementary material for: Comparative Genomics of Flowering Time Pathways Using Brachypodium distachyon as a Model for the Temperate Grasses
Source: PLoS One. 2010 Apr 19;5(4):e10065. doi: 10.1371/journal.pone.0010065 (PMC2856676; doi:10.1371/journal.pone.0010065)
Supplement: Figure S4 — The relationship between Arabidopsis CIB1 and other proteins in subgroup 12 of the bHLH family. The region of the alignment used to estimate the tree spanned the bHLH domain and adjacent regions that were also conserved in proteins belonging to this subgroup. Neither bootstrap analysis nor studying regions outside this region provided evidence for an orthologue of Arabidopsis CIB1 in Brachypodium. (0.08 MB PPT) [file pone.0010065.s005.ppt]

## Slide 1
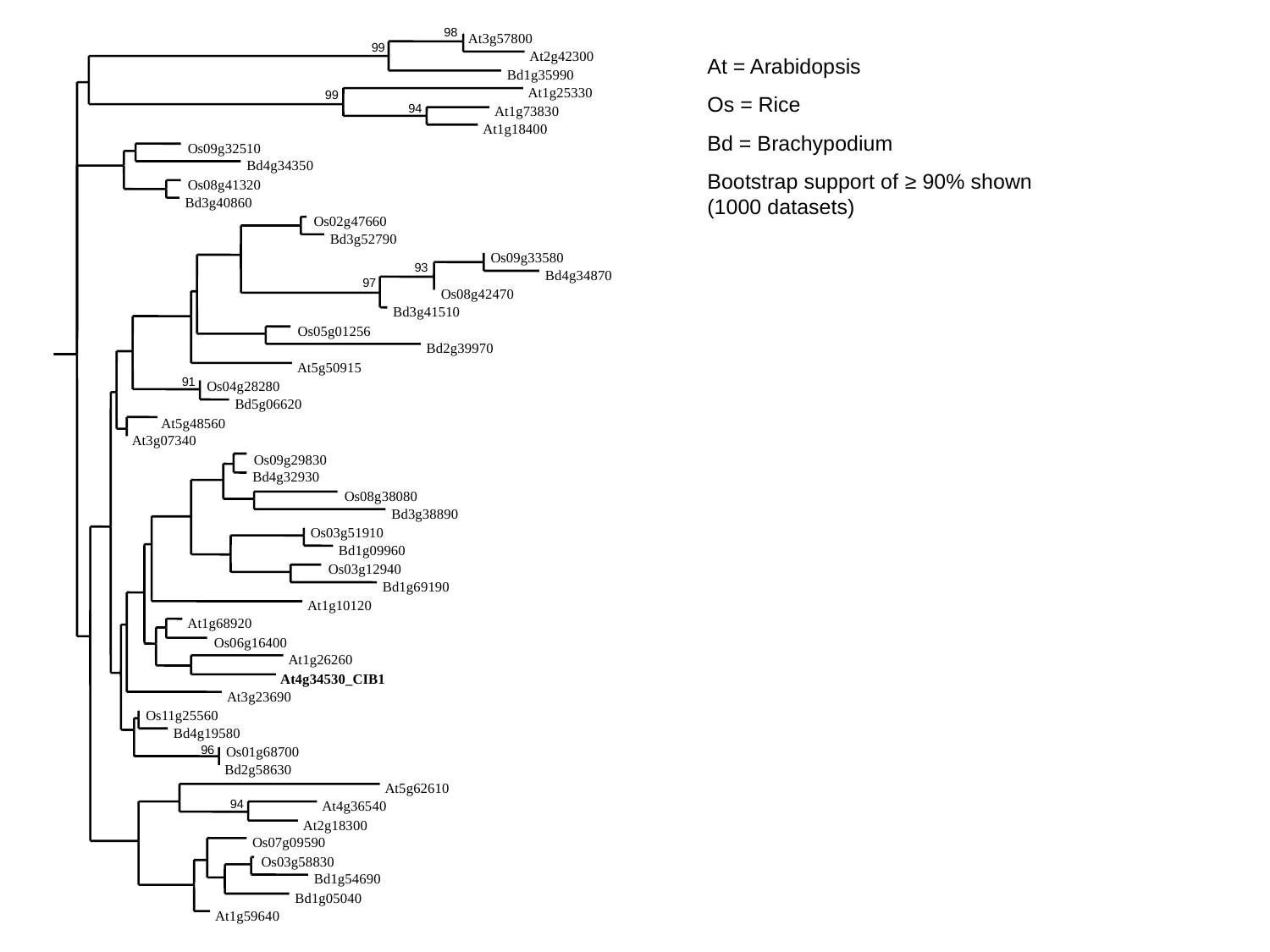

98
At3g57800
At2g42300
Bd1g35990
At1g25330
At1g73830
At1g18400
Os09g32510
Bd4g34350
Os08g41320
Bd3g40860
Os02g47660
Bd3g52790
Os09g33580
Bd4g34870
Os08g42470
Bd3g41510
Os05g01256
Bd2g39970
At5g50915
Os04g28280
Bd5g06620
At5g48560
At3g07340
Os09g29830
Bd4g32930
Os08g38080
Bd3g38890
Os03g51910
Bd1g09960
Os03g12940
Bd1g69190
At1g10120
At1g68920
Os06g16400
At1g26260
At4g34530_CIB1
At3g23690
Os11g25560
Bd4g19580
Os01g68700
Bd2g58630
At5g62610
At4g36540
At2g18300
Os07g09590
Os03g58830
Bd1g54690
Bd1g05040
At1g59640
99
At = Arabidopsis
Os = Rice
Bd = Brachypodium
Bootstrap support of ≥ 90% shown
(1000 datasets)
99
94
93
97
91
96
94
