# Supplementary material for: Comparative Genomics of Flowering Time Pathways Using Brachypodium distachyon as a Model for the Temperate Grasses
Source: PLoS One. 2010 Apr 19;5(4):e10065. doi: 10.1371/journal.pone.0010065 (PMC2856676; doi:10.1371/journal.pone.0010065)
Supplement: Figure S5 — The relationship between members of the NF-YB-like (HAP) family. A conserved region of eighty four amino acids was identified in an alignment of these proteins and used to estimate the tree. For this family, the alignment was created by aligning the sequences to a profile HMM of the conserved region. (0.11 MB PPT) [file pone.0010065.s006.ppt]

## Slide 1
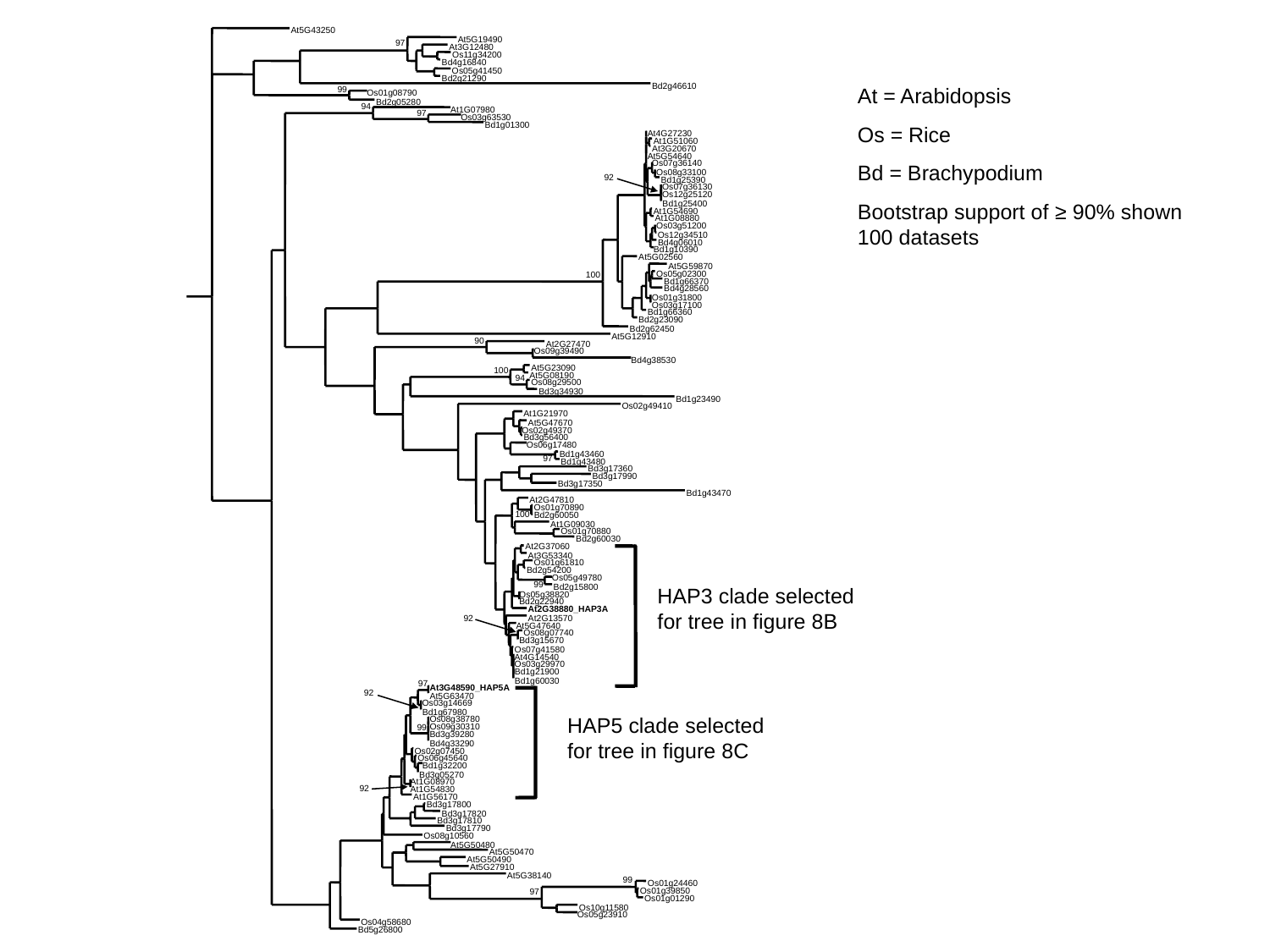

At5G43250
At5G19490
At3G12480
Os11g34200
Bd4g16840
Os05g41450
Bd2g21290
Bd2g46610
Os01g08790
Bd2g05280
At1G07980
Os03g63530
Bd1g01300
At4G27230
At1G51060
At3G20670
At5G54640
Os07g36140
Os08g33100
Bd1g25390
Os07g36130
Os12g25120
Bd1g25400
At1G54690
At1G08880
Os03g51200
Os12g34510
Bd4g06010
Bd1g10390
At5G02560
At5G59870
Os05g02300
Bd1g66370
Bd4g28560
Os01g31800
Os03g17100
Bd1g66360
Bd2g23090
Bd2g62450
At5G12910
At2G27470
Os09g39490
Bd4g38530
At5G23090
At5G08190
Os08g29500
Bd3g34930
Bd1g23490
Os02g49410
At1G21970
At5G47670
Os02g49370
Bd3g56400
Os06g17480
Bd1g43460
Bd1g43480
Bd3g17360
Bd3g17990
Bd3g17350
Bd1g43470
At2G47810
Os01g70890
Bd2g60050
At1G09030
Os01g70880
Bd2g60030
At2G37060
At3G53340
Os01g61810
Bd2g54200
Os05g49780
Bd2g15800
Os05g38820
Bd2g22940
At2G38880_HAP3A
At2G13570
At5G47640
Os08g07740
Bd3g15670
Os07g41580
At4G14540
Os03g29970
Bd1g21900
Bd1g60030
At3G48590_HAP5A
At5G63470
Os03g14669
Bd1g67980
Os08g38780
Os09g30310
Bd3g39280
Bd4g33290
Os02g07450
Os06g45640
Bd1g32200
Bd3g05270
At1G08970
At1G54830
At1G56170
Bd3g17800
Bd3g17820
Bd3g17810
Bd3g17790
Os08g10560
At5G50480
At5G50470
At5G50490
At5G27910
At5G38140
Os01g24460
Os01g39850
Os01g01290
Os10g11580
Os05g23910
Os04g58680
Bd5g26800
97
At = Arabidopsis
Os = Rice
Bd = Brachypodium
Bootstrap support of ≥ 90% shown
100 datasets
99
94
97
92
100
90
100
94
97
100
99
HAP3 clade selected for tree in figure 8B
92
97
92
HAP5 clade selected for tree in figure 8C
99
92
99
97
