# Supplementary material for: Comparative Genomics of Flowering Time Pathways Using Brachypodium distachyon as a Model for the Temperate Grasses
Source: PLoS One. 2010 Apr 19;5(4):e10065. doi: 10.1371/journal.pone.0010065 (PMC2856676; doi:10.1371/journal.pone.0010065)
Supplement: Figure S6 — The relationship between the rice GF14c protein and the rest of the 14-3-3 protein family. The full alignment was used to estimate the tree, except that the alignment ends were trimmed. (0.07 MB PPT) [file pone.0010065.s007.ppt]

## Slide 1
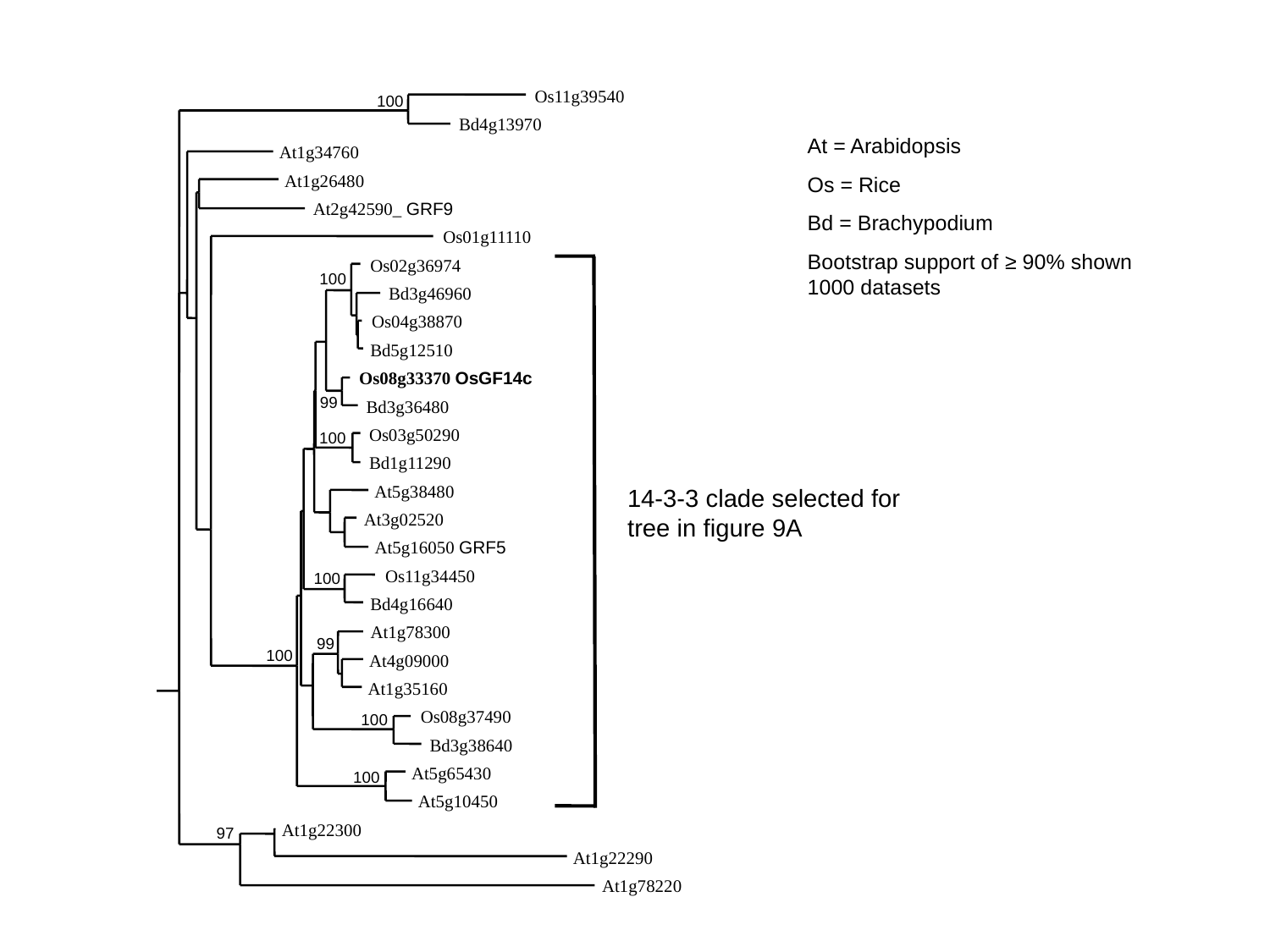

100
Os11g39540
Bd4g13970
At1g34760
At1g26480
At2g42590_ GRF9
Os01g11110
Os02g36974
Bd3g46960
Os04g38870
Bd5g12510
Os08g33370 OsGF14c
Bd3g36480
Os03g50290
Bd1g11290
At5g38480
At3g02520
At5g16050 GRF5
Os11g34450
Bd4g16640
At1g78300
At4g09000
At1g35160
Os08g37490
Bd3g38640
At5g65430
At5g10450
At1g22300
At1g22290
At1g78220
At = Arabidopsis
Os = Rice
Bd = Brachypodium
Bootstrap support of ≥ 90% shown
1000 datasets
100
99
100
14-3-3 clade selected for tree in figure 9A
100
99
100
100
100
97
