# Supplementary material for: Comparative Genomics of Flowering Time Pathways Using Brachypodium distachyon as a Model for the Temperate Grasses
Source: PLoS One. 2010 Apr 19;5(4):e10065. doi: 10.1371/journal.pone.0010065 (PMC2856676; doi:10.1371/journal.pone.0010065)
Supplement: Figure S7 — The relationship between flowering time genes that belong to the MADS-box family. The alignment was created by aligning all the sequences corresponding to this family to a profile HMM of the MADS-box domain. Nineteen poorly aligned proteins were removed from the data set before estimating the tree. (0.17 MB PPT) [file pone.0010065.s008.ppt]

## Slide 1
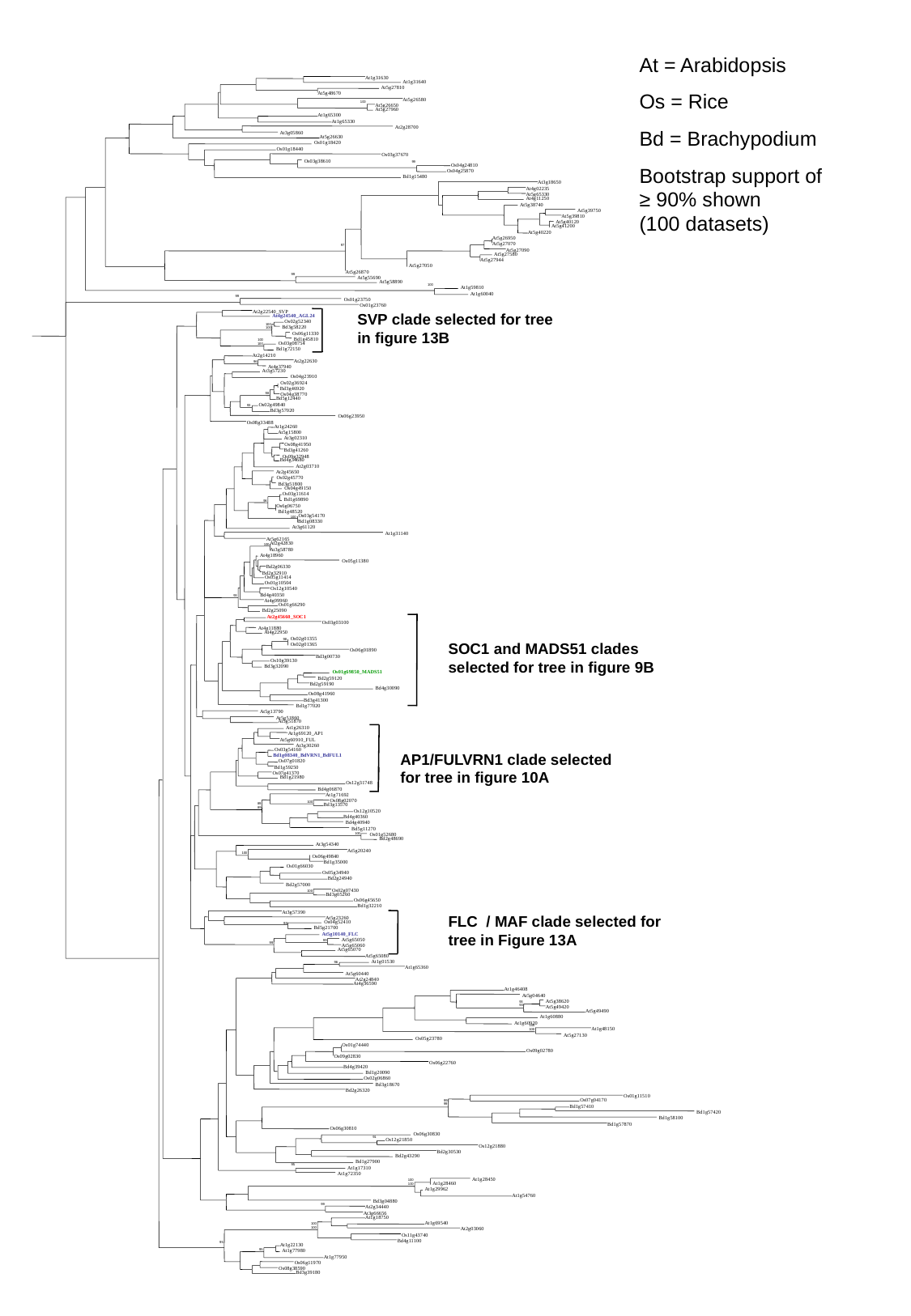

At = Arabidopsis
Os = Rice
Bd = Brachypodium
Bootstrap support of ≥ 90% shown
(100 datasets)
At1g31630
At1g31640
At5g27810
At5g48670
At5g26580
At5g26650
At5g27960
At1g65300
At1g65330
At2g28700
At3g05860
At5g26630
Os01g18420
Os01g18440
Os03g37670
Os03g38610
Os04g24810
Os04g25870
Bd1g15480
At3g18650
At4g02235
At5g65330
At4g11250
At5g38740
At5g39750
At5g39810
At5g40120
At5g41200
At5g40220
At5g26950
At5g27070
At5g27090
At5g27580
At5g27944
At5g27050
At5g26870
At5g55690
At5g58890
At1g59810
At1g60040
Os01g23750
Os01g23760
At2g22540_SVP
At4g24540_AGL24
Os02g52340
Bd3g58220
Os06g11330
Bd1g45810
Os03g08754
Bd1g72150
At2g14210
At2g22630
At4g37940
At3g57230
Os04g23910
Os02g36924
Bd3g46920
Os04g38770
Bd5g12440
Os02g49840
Bd3g57020
Os06g23950
Os08g33488
At1g24260
At5g15800
At3g02310
Os08g41950
Bd3g41260
Os09g32948
Bd4g34680
At2g03710
At2g45650
Os02g45770
Bd3g51800
Os04g49150
Os03g11614
Bd1g69890
Os6g06750
Bd1g48520
Os03g54170
Bd1g08330
At3g61120
At1g31140
At5g62165
At2g42830
At3g58780
At4g18960
Os05g11380
Bd2g06330
Bd2g32910
Os05g11414
Os01g10504
Os12g10540
Bd4g40350
At4g09960
Os01g66290
Bd2g25090
At2g45660_SOC1
Os03g03100
At4g11880
At4g22950
Os02g01355
Os02g01365
Os06g01890
Bd3g00730
Os10g39130
Bd3g32090
Os01g69850_MADS51
Bd2g59120
Bd2g59190
Bd4g30090
Os08g41960
Bd3g41300
Bd1g77020
At5g13790
At5g51860
At5g51870
At1g26310
At1g69120_AP1
At5g60910_FUL
At3g30260
Os03g54160
Bd1g08340_BdVRN1_BdFUL1
Os07g01820
Bd1g59250
Os07g41370
Bd1g21980
Os12g31748
Bd4g06870
At1g71692
Os08g02070
Bd3g13570
Os12g10520
Bd4g40360
Bd4g40940
Bd5g11270
Os01g52680
Bd2g48690
At3g54340
At5g20240
Os06g49840
Bd1g35000
Os01g66030
Os05g34940
Bd2g24940
Bd2g57000
Os02g07430
Bd3g05260
Os06g45650
Bd1g32210
At3g57390
At5g23260
Os04g52410
Bd5g21700
At5g10140_FLC
At5g65050
At5g65060
At5g65070
At5g65080
At1g01530
At1g65360
At5g60440
At2g24840
At4g36590
At1g46408
At5g04640
At5g38620
At5g49420
At5g49490
At1g60880
At1g60920
At1g48150
At5g27130
Os05g23780
Os01g74440
Os09g02780
Os09g02830
Os06g22760
Bd4g39420
Bd1g20090
Os02g06860
Bd3g18670
Bd2g26320
Os01g11510
Os07g04170
Bd1g57410
Bd1g57420
Bd1g58100
Bd1g57870
Os06g30810
Os06g30830
Os12g21850
Os12g21880
Bd2g30530
Bd2g43290
Bd1g27900
At1g17310
At1g72350
At1g28450
At1g28460
At1g29962
At1g54760
Bd3g04880
At2g34440
At3g66656
At1g18750
At1g69540
At2g03060
Os11g43740
Bd4g11100
At1g22130
At1g77980
At1g77950
Os06g11970
Os08g38590
Bd3g39180
100
99
97
99
100
99
SVP clade selected for tree in figure 13B
100
100
100
100
96
98
93
96
100
100
93
98
SOC1 and MADS51 clades selected for tree in figure 9B
AP1/FULVRN1 clade selected for tree in figure 10A
100
89
89
100
100
100
FLC / MAF clade selected for tree in Figure 13A
98
99
99
96
93
93
100
100
99
99
91
95
100
100
99
100
100
95
95
