# Supplementary material for: Comparative Genomics of Flowering Time Pathways Using Brachypodium distachyon as a Model for the Temperate Grasses
Source: PLoS One. 2010 Apr 19;5(4):e10065. doi: 10.1371/journal.pone.0010065 (PMC2856676; doi:10.1371/journal.pone.0010065)
Supplement: Figure S8 — The relationship between SPL proteins. The region of the alignment used to estimate the tree corresponded to the pFAM profile HMM (PF03110) but excluded columns containing non-homologous amino acids. (0.08 MB PPT) [file pone.0010065.s009.ppt]

## Slide 1
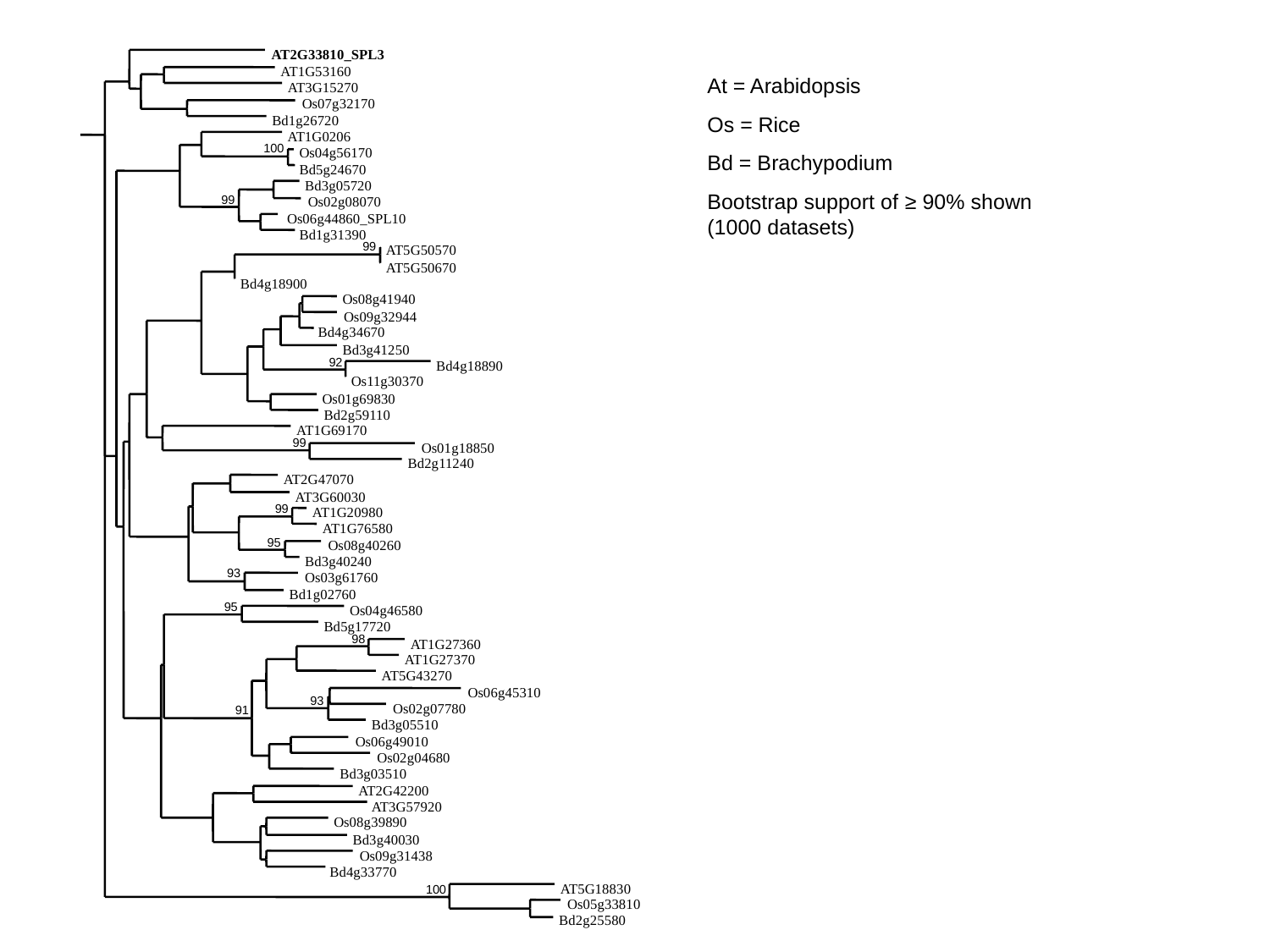

AT2G33810_SPL3
AT1G53160
AT3G15270
Os07g32170
Bd1g26720
AT1G0206
Os04g56170
Bd5g24670
Bd3g05720
Os02g08070
Os06g44860_SPL10
Bd1g31390
AT5G50570
AT5G50670
Bd4g18900
Os08g41940
Os09g32944
Bd4g34670
Bd3g41250
Bd4g18890
Os11g30370
Os01g69830
Bd2g59110
AT1G69170
Os01g18850
Bd2g11240
AT2G47070
AT3G60030
AT1G20980
AT1G76580
Os08g40260
Bd3g40240
Os03g61760
Bd1g02760
Os04g46580
Bd5g17720
AT1G27360
AT1G27370
AT5G43270
Os06g45310
Os02g07780
Bd3g05510
Os06g49010
Os02g04680
Bd3g03510
AT2G42200
AT3G57920
Os08g39890
Bd3g40030
Os09g31438
Bd4g33770
AT5G18830
Os05g33810
Bd2g25580
At = Arabidopsis
Os = Rice
Bd = Brachypodium
Bootstrap support of ≥ 90% shown
(1000 datasets)
100
99
99
92
99
99
95
93
95
98
93
91
100
