# Supplementary material for: Comparative Genomics of Flowering Time Pathways Using Brachypodium distachyon as a Model for the Temperate Grasses
Source: PLoS One. 2010 Apr 19;5(4):e10065. doi: 10.1371/journal.pone.0010065 (PMC2856676; doi:10.1371/journal.pone.0010065)
Supplement: Figure S9 — The relationship between the FRIGIDA protein and related proteins. The region of the alignment used to estimate the tree corresponded to the pFAM profile HMM (PF07899) but excluded columns containing non-homologous amino acids. (0.07 MB PPT) [file pone.0010065.s010.ppt]

## Slide 1
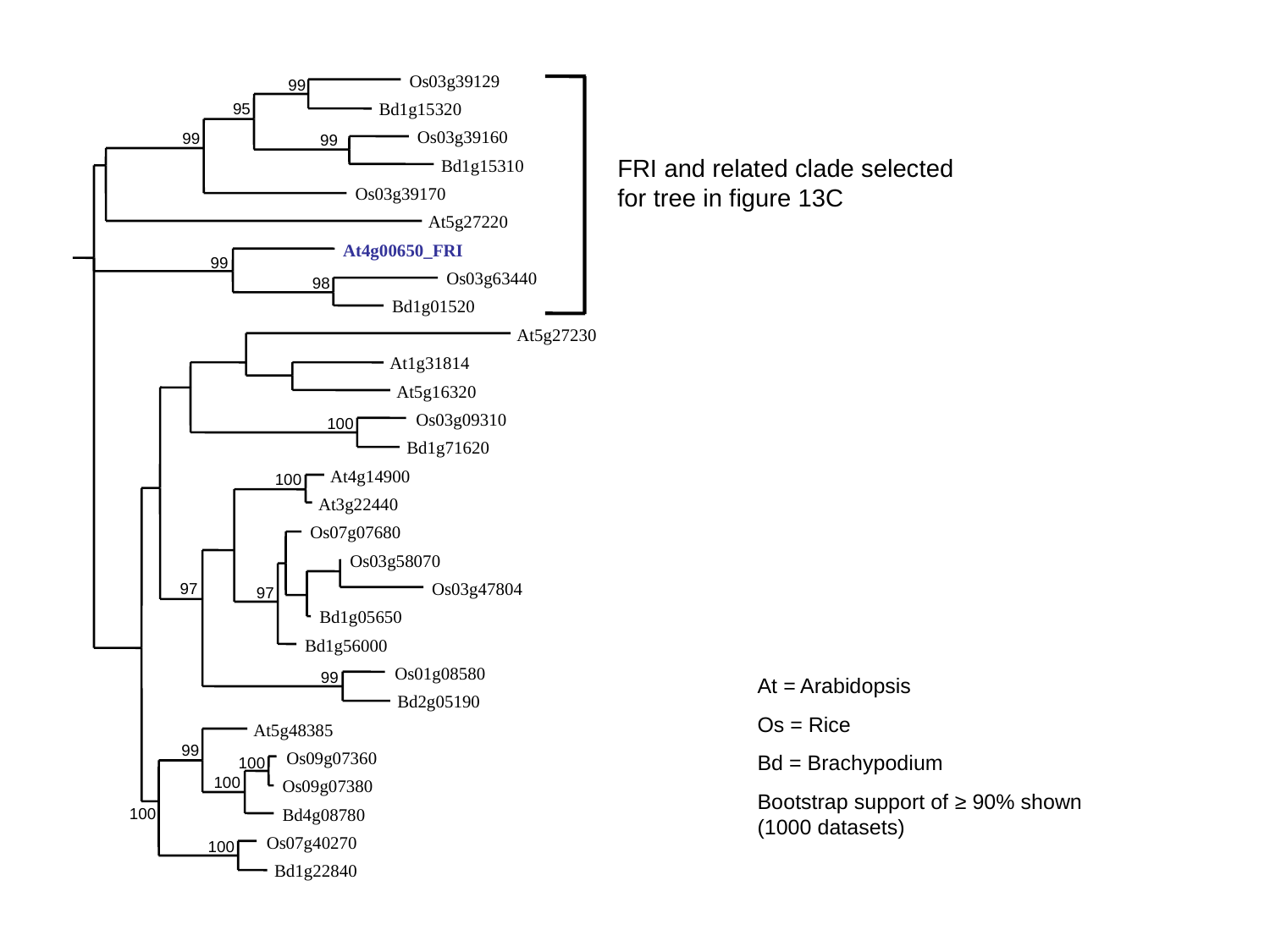

99
Os03g39129
Bd1g15320
Os03g39160
Bd1g15310
Os03g39170
At5g27220
At4g00650_FRI
Os03g63440
Bd1g01520
At5g27230
At1g31814
At5g16320
Os03g09310
Bd1g71620
At4g14900
At3g22440
Os07g07680
Os03g58070
Os03g47804
Bd1g05650
Bd1g56000
Os01g08580
Bd2g05190
At5g48385
Os09g07360
Os09g07380
Bd4g08780
Os07g40270
Bd1g22840
95
99
99
FRI and related clade selected for tree in figure 13C
99
98
100
100
97
97
99
At = Arabidopsis
Os = Rice
Bd = Brachypodium
Bootstrap support of ≥ 90% shown
(1000 datasets)
99
100
100
100
100
